# Accurate prediction of molecular targets using a self-supervised image representation learning framework

**DOI:** 10.21203/rs.3.rs-1477870/v1

**Published:** 2022-04-07

**Authors:** Xiangxiang Zeng, Hongxin Xiang, Linhui Yu, Jianmin Wang, Kenli Li, Ruth Nussinov, Feixiong Cheng

**Affiliations:** 1 College of Computer Science and Electronic Engineering, Hunan University, Changsha, Hunan, 410082, China; 2 Computational Structural Biology Section, Frederick National Laboratory for Cancer Research in the Laboratory of Cancer Immunometabolism, National Cancer Institute, Frederick, MD 21702, USA; 3 Department of Human Molecular Genetics and Biochemistry, Sackler School of Medicine, Tel Aviv University, Tel Aviv 69978, Israel; 4 Genomic Medicine Institute, Lerner Research Institute, Cleveland Clinic, Cleveland, OH 44195, USA; 5 Department of Molecular Medicine, Cleveland Clinic Lerner College of Medicine, Case Western Reserve University, Cleveland, OH 44195, USA; 6 Case Comprehensive Cancer Center, Case Western Reserve University School of Medicine, Cleveland, OH 44106, USA

## Abstract

The clinical efficacy and safety of a drug is determined by its molecular targets in the human proteome. However, proteome-wide evaluation of all compounds in human, or even animal models, is challenging. In this study, we present an unsupervised pre-training deep learning framework, termed ImageMol, from 8.5 million unlabeled drug-like molecules to predict molecular targets of candidate compounds. The ImageMol framework is designed to pretrain chemical representations from unlabeled molecular images based on local- and global-structural characteristics of molecules from pixels. We demonstrate high performance of ImageMol in evaluation of molecular properties (i.e., drug’s metabolism, brain penetration and toxicity) and molecular target profiles (i.e., human immunodeficiency virus) across 10 benchmark datasets. ImageMol shows high accuracy in identifying anti-SARS-CoV-2 molecules across 13 high-throughput experimental datasets from the National Center for Advancing Translational Sciences (NCATS) and we re-prioritized candidate clinical 3CL inhibitors for potential treatment of COVID-19. In summary, ImageMol is an active self-supervised image processing-based strategy that offers a powerful toolbox for computational drug discovery in a variety of human diseases, including COVID-19.

## Introduction

Despite recent advances of biomedical research and technologies, drug discovery and development remains a challenging multidimensional task requiring optimization of vital properties of candidate compounds, including pharmacokinetics, efficacy and safety [1, 2]. It was estimated that pharmaceutical companies spent $2.6 billion in 2015, up from $802 million in 2003, on drug approval by the U.S. Food and Drug Administration (FDA) [3]. The increasing cost of drug development resulted from lack of efficacy of the randomized controlled trials, and the unknown pharmacokinetics and safety profiles of candidate compounds [4–6]. Traditional experimental approaches are unfeasible on proteome-wide scale evaluation of molecular targets for all candidate compounds in human, or even animal models. Computational approaches and technologies have been considered a promising solution [7, 8], which can significantly reduce costs and time during the entire pipeline of the drug discovery and development.

The rise of advanced Artificial Intelligence (AI) technologies [9, 10], motivated their application to drug design [11–13] and target identification [14–16]. One of the fundamental challenges is how to learn molecular representation from chemical structures [17]. Previous molecular representations were based on hand-crafted features, such as fingerprint-based features [16, 18], physiochemical descriptors and pharmacophore-based features [19, 20]. However, these traditional molecular representation methods rely on a large amount of domain knowledge, such as sequence-based [21, 22] and graph-based [23, 24] approaches. Their accuracy in extracting informative vectors for description of molecular identities and biological characteristics of the molecules is limited. Recent advances of unsupervised learning in computer vision [25, 26] suggest that it is possible to apply unsupervised image-based pre-training models for computational drug discovery.

In this study, we presented an unsupervised molecular image pretraining framework (termed ImageMol) with chemical awareness for learning the molecular structures from large-scale molecular images. ImageMol combines an image processing framework with comprehensive molecular chemistry knowledge for extracting fine pixel-level molecular features in a visual computing way. Compared with state-of-the-art methods, ImageMol has two significant improvements: (1) It utilizes molecular images as the feature representation of compounds with high accuracy and low computing cost; (2) It exploits an unsupervised pre-trained learning framework to capture the structural information of molecular images from 8.5 million drug-like compounds with diverse biological activities at the human proteome (Figure 1). We demonstrated the high accuracy of ImageMol in a variety of drug discovery tasks. Via ImageMol, we identified anti-SARS-CoV-2 molecules across 13 high-throughput experimental datasets from the National Center for Advancing Translational Sciences (NCATS). In summary, ImageMol provides a powerful pre-training deep learning framework for computational drug discovery.

## Results

### Description of ImageMol

Here, we developed a pre-training deep learning framework, ImageMol, for accurate prediction of molecular targets. ImageMol pre-trained 8,506,205 molecular images from two large drug-like databases (ChEMBL [27] and ZINC [28]). We assembled five pretext tasks to extract biologically relevant structural information: 1) A molecular encoder was designed to extract latent features from 8.5 million molecular images (Fig. 1a); 2) Five pretraining strategies (Supplementary Figures 1–5) are utilized to optimize the latent representation of the molecular encoder by considering the chemical knowledge and structural information from molecular images (Fig. 1b); and 3) a pretrained molecular encoder is further fine-tuned on downstream tasks to further improve model performance (Fig. 1c). In addition, two pre-tasks (multi-granularity chemical clusters classification task and molecular rationality discrimination task (*cf*. Methods) are further designed to ensure that ImageMol properly capture meaningful chemical information from images (Supplementary Figures 1–2). We next evaluated the performance of ImageMol in a variety of drug discovery tasks, including evaluation of the drug’s metabolism, brain penetration, toxic profiles, and molecular target profiles across the human immunodeficiency virus (HIV), SARS-CoV-2, and Alzheimer’s disease.

### Benchmark evaluation of ImageMol

We first evaluated the performance of ImageMol using four types of benchmark datasets (Supplementary Tables 1 and 2): (1) molecular targets (human immunodeficiency virus [HIV] and beta-secretase [BACE, a key target in Alzheimer’s disease]); (2) blood-brain barrier penetration (BBBP); (3) drug’s metabolism, and (4) molecular Toxicity using the 21st century (Tox21) and clinical trial toxicity (ClinTox) databases (*cf*. Methods).

Using the area under the curve (AUC) under the receiver operating characteristic (ROC) curve, ImageMol achieves high AUC values (Fig. 2a) across HIV (AUC=0.821), BACE (AUC=0.902), BBBP (AUC=0.931), Tox21 (AUC=0.809). In stratified split, the proportion of each class in the training set, validation set, and test set is the same as in the original dataset. In scaffold split, the datasets are divided according to the molecular substructure: the substructures in the training set, validation set, and test set are disjoint, making them ideal to test robustness and generalizability of *in silico* models. For a fair comparison in Fig. 2b, we used the same experimental setup as Chemception [29], a state-of-the-art convolutional neural network (CNN) framework. ImageMol achieves higher AUC values on HIV (AUC=0.821) and Tox21 (AUC=0.824), suggesting that ImageMol can capture more biologically relevant information from molecular images than CNN. We further evaluated performance of ImageMol in prediction of drug metabolism across five major metabolism enzymes: CYP1A2, CYP2C9, CYP2C19, CYP2D6 and CYP3A4 (*cf*. Methods). Figure 2c showed that ImageMol achieves higher AUC values (ranging from 0.802 to 0.892) in the prediction of inhibitors vs. non-inhibitors across five major drug metabolism enzymes as well compared with two state-of-the-art molecular image-based representation models: ADMET-CNN [30] and QSAR-CNN [31]. Additional results of the detailed comparison are provided in the Supplementary Figures 6–7.

We further compared the performance of ImageMol with three state-of-the-art molecular representation models: 1) fingerprint-based, 2) sequence-based, and 3) graph-based models. As shown in Fig. 2d, ImageMol outperforms two sequence-based models (SMILES Transformer [22] and Recurrent Neural Network-based Sequence-to-Sequence (RNNS2S) [32]) across all four benchmark biomedical datasets with stratified split. ImageMol has better performance (Fig. 2e) compared with three sequence-based models (ChemBERTa [21], SMILES Transformer and Mol2Vec [33]) and two graph-based models (Jure’s GNN [23] and N-GRAM [34]) based on a scaffold split. In addition, we found that ImageMol achieved higher AUC values (Fig. 2f) compared to traditional MACCS-based methods and FP4-based methods [35] across multiple machine learning algorithms, including support vector machine, Decision Tree, k-Nearest Neighbors, Naive Bayes (NB), and their ensemble models [35] (Supplementary Table 3). The detailed comparisons of ImageMol with each model/method are provided in the Supplemental Methods and Results. Altogether, ImageMol achieves high performance in multiple drug discovery tasks, outperforming state-of-the-art methods (Fig. 2d–2f and Supplementary Tables 4–5).

### Prediction of anti-viral activities across 13 SARS-CoV-2 targets

The ongoing global COVID-19 pandemic caused by a toxic agent SARS-CoV-2 virus, has led to more than 1.1 billion confirmed cases and over 6 million deaths worldwide as of March 15, 2022. There is a critical, time-sensitive need to develop effective anti-viral treatment strategies for the COVID-19 pandemic [6, 36]. We therefore test our ImageMol to identify potential anti-SARS-CoV-2 treatments across a variety of SARS-CoV-2 biological assays, including viral replication, viral entry, counterscreen, in vitro infectivity, and live virus infectivity [20]. In total, we evaluated ImageMol across 13 SARS-CoV-2 datasets, including 3C-like (3CL) protease enzymatic activity, angiotensin converting enzyme 2 (ACE2) enzymatic activity, human embryonic kidney 293 (KEK293) cell line toxicity, human fibroblast toxicity (Hcytox), middle east respiratory syndrome pseudotyped particle entry (MERS-PPE) and its Huh7 tox counterscreen (MERS-PPE_cs), SARS-CoV PPE (CoV-PPE) and its VeroE6 tox counterscreen (CoV-PPE_cs), SARS-CoV-2 cytopathic effect (CPE) and its host tox counterscreen (Cytotox), Spike-ACE2 protein-protein interaction (AlphaLISA) and its TruHit counterscreen, and transmembrane protease serine 2 (TMPRSS2) enzymatic activity (Supplementary Table 6).

Across 13 SARS-CoV-2 targets, ImageMol achieves high AUC values ranging from 72.0% to 82.6% (Fig. 3a). To test whether ImageMol capture biologically relevant features, we used the global average pooling (GAP) layer of ImageMol to extract latent features of each dataset and used t-SNE to visualize latent features. Fig. 3b revealed that the latent features identified by ImageMol are well clustered according to whether they are active or inactive anti-SARS-CoV-2 agents across 8 targets or endpoints. These observations showed that ImageMol can accurately extract discriminative, antiviral features from molecular images for downstream tasks.

We further compared ImageMol with both deep learning and machine learning frameworks: 1) a graph neural network (GNN) with a series of pretraining strategies (termed Jure’s GNN [23]), and (2) REDIAL-2020 [20], a suite of machine learning models for estimating small molecule activities in a range of SARS-CoV-2-related assays. We found that ImageMol significantly outperform Jure’s GNN models across all 13 SARS-CoV-2 targets (Fig. 3a and Supplementary Table 7). For instance, there are over 12% elevated AUC values of ImageMol (AUC = 0.824) compared to the Jure’s GNN model (AUC = 0.704) in prediction of 3CL protease inhibitors. We further evaluated the area under the precision and recall (AUPR), a metric that is highly sensitive to the imbalance issues of positive versus negative labeled data. Compared to Jure’s GNN models, the elevated AUPR of ImageMol ranges from 3.0% to 29.1% with an average performance advantage of 8.5% across 13 SARS-CoV-2 targets, in particular for 3CL protease inhibitors (29.1% AUPR improvement) and ACE2 enzymatic activities (26.9% APUR improvement). To compared with REDIAL-2020 [20], we used the same experimental settings and the performance evaluation metrics, including accuracy, sensitivity, precision, F1 (the harmonic mean between sensitivity and precision) and AUC. We found that ImageMol outperformed REDIAL-2020 as well (Supplementary Table 8).

In summary, these comprehensive evaluations reveal high accuracy of ImageMol in identifying anti-SARS-CoV-2 molecules across diverse viral targets and phenotypic assays. Furthermore, ImageMol is more capable on datasets with extreme imbalance of positive and negative samples compared to traditional deep learning pre-trained models [23] or machine learning approaches [20].

### Identifying anti-SARS-CoV-2 inhibitors via ImageMol

We next turned to identify potential anti-SARS-CoV-2 inhibitors using 3CL protease as a prototypical example as it has been shown a promising target for therapeutic development in treating of COVID-19 [37, 38]. We focused on 2,501 U.S. FDA-approved drugs from DrugBank [39] to identify ImageMol-predicted 3CL protease inhibitors as repurposable drugs for COVID-19 using a drug repurposing strategy [36].

Via molecular image representation of 3CL protease inhibitor vs. non-inhibitor dataset under the ImageMol framework, we found that 3CL inhibitors and non-inhibitors are well separated in a t-distributed Stochastic Neighbor Embedding (t-SNE) plot (Fig. 3c). Molecules with activity concentration 50% (AC_50_) less than 10 uM were defined as inhibitors, otherwise they were non-inhibitors. We showed the probability of each drug in DrugBank being inferred as a 3CL protease inhibitor (Supplementary Table 9) and visualized their overall probability distribution (Supplementary Figure 8). We found that 12 of the top 20 drugs (60%) have been validated (including cell assay, clinical trial, etc.) as potential SARS-CoV-2 inhibitors (Supplementary Table 9), among which 3 drugs are further verified as potential 3CL protease inhibitors by biological experiments (Fig. 3d). To test the generalization ability of ImageMol, we used 10 experimentally reported 3CL protease inhibitors as an external validation set (Supplementary Table 10). ImageMol identified 6 out of 10 known 3CL protease inhibitors (60% success rate, Fig. 3e), suggesting a high generalization ability in anti-SARS-CoV-2 drug discovery.

We further used the HEY293 assay to predict anti-SARS-CoV-2 repurposable drugs. We collected experimental evidence for top 20 drugs as potential SARS-CoV-2 inhibitors (Supplementary Table 11). We found that 13 out of 20 drugs (65%) have been validated by different experimental assays as potential inhibitors for the treatment of SARS-CoV-2 (such as *in vitro* cellular assays and clinical trials) in Supplementary Table 11.

Meanwhile, 122 drugs have been identified to block SARS-CoV-2 infection [40]. From these drugs, we selected a total of 70 small molecules overlapped in DrugBank to evaluate performance of the KEY293 model. We found that ImageMol successfully predicted 47 out of 70 (67.1% success rate, Supplementary Table 12), suggesting a high generalizability of ImageMol for inferring potential candidate drugs in the HEY293 assay as well.

### Biological Interpretation of ImageMol

We next turned to use t-SNE to visualize molecular representations from different models to test the biological interpretation of ImageMol. We used the clusters identified by the multi-granularity chemical clusters classification (MG3C) task (*cf*. Methods) to split the molecular structures. We randomly selected 10% clusters obtained from MG3C and sampled 1,000 molecules for each cluster. We performed three comparisons for each molecule: a) MACCS fingerprints with 166-dimensional (166D) features, b) ImageMol without pretrained models with 512D features, and c) ImageMol pre-trained 512D features. We found that ImageMol distinguish molecular structures very well (Fig. 4e and Supplementary Figure 9c), outperforming that of MACCS fingerprints (Supplementary Figure 9a) and non-pre-trained models (Supplementary Figure 9b). ImageMol can capture priori knowledge of chemical information from the molecular image representations, including =O bond, −OH bond, −NH3 bond and benzene ring (Fig. 4a). We further used the Davies Bouldin (DB) index [34] to quantitatively evaluate the clustering results and the smaller DB index represents the better performance. We found that ImageMol (DB index=1.98) was better than MACCS fingerprint (DB index=2.13); furthermore, pre-trained models can significantly improve the molecular representation as well (DB index=18.48).

Gradient-weighted Class Activation Mapping (Grad-CAM) [41] is a commonly used convolutional neural network (CNN) visualization method [42, 43]. Figures 4b and 4c illustrate 12 example molecules of the Grad-CAM visualization of ImageMol (*cf*. Supplementary Figures 10 and 11). ImageMol accurately captures attention to the global (Fig. 4b) and the local (Fig. 4c) structural information simultaneously. In addition, we counted the proportion of blank areas in the images to the entire molecular image across all 13 SARS-CoV-2 datasets (Supplementary Table 13). We found an average sparsity (sparsity refers to the proportion of blank areas in an image) of 94.9% across the entire dataset, suggesting that ImageMol models are easily inclined to use blank areas of the image for meaningless inferences [31]. Figure 4d shows that ImageMol primarily pays attention to the middle area of the image during predictions. Thus, ImageMol indeed predicts based on the molecular structures rather than uses meaningless blank areas. We further calculated the coarse-grained and fine-grained hit rates (Supplementary Figure 12). The coarse-grained hit rate illustrates that ImageMol can utilize molecular structures of all images for inference, with a ratio of 100%, compared to the QSAR-CNN models [31] with 90.7%. The fine-grained hit rate shows that ImageMol can leverage almost all structural information in molecular images to inference, with a ratio of over 99%, reflecting its ability to capture global information of molecules.

In summary, ImageMol captures the biologically relevant chemical information of molecular images with both local- and global-levels of structural information, outperforming existing state-of-the-art deep learning approaches (Fig. 4).

### 2.6 Ablation analysis of ImageMol

The robustness of the model to hyperparameter tuning is important because the initialization of different parameters can affect the performance of the model [44]. Here, we explore the impact of pre-training strategies on the hyperparameter tuning of ImageMol. As shown in Supplementary Tables 4–5, ImageMol is more robust than ImageMol_NonPretrained, with an average performance variance of 1.2% versus 2.4%. Therefore, pre-training strategies improve the robustness of ImageMol to initialization parameters.

To explore the impact of pre-training with different data scales, we first use 0 million (no pre-training), 0.2 million, 0.6 million, 1 million, and 8.5 million drug-like compounds to pretrain ImageMol respectively and then evaluate their performance. We found that the average ROC-AUC performance of 0 million (75.7%), 0.2 million (76.9%), 0.6 million (81.6%), 1 million (83.8%) and 8.5 million (85.9%) increased from 1.2% to 10.2% as the pre-trained data size increases. Thus, ImageMol can be further improved as the more drug-like molecules cancer be pre-trained. We further investigated the impact of different pretext tasks using multi-granularity chemical clusters classification (MG3C), jigsaw puzzle prediction (JPP), and MASK-based contrastive learning (MCL) (*cf*. Methods), respectively. We found that each pretext task improves the mean AUC value of ImageMol from 0.7% to 4.9%: without pretext task (75.7%), JPP (78.8%), MG3C (80.6%]) and MCL (76.4%) (Supplementary Figure 14). The best performance was achieved by assembling all 3 pretext tasks for pre-training (AUC = 85.9%, Supplementary Figure 14). In summary, each task integrated implemented the ImageMol framework synergistically improve performance and models can be improved further by hyperparameter tuning and pre-training from a bigger drug-like chemical datasets in the future.

## Discussion

We presented a self-supervised image processing-based pre-training deep learning framework that combines molecular images and unsupervised learning to learn molecular representations. We demonstrated the high accuracy of ImageMol across multiple benchmark biomedical datasets with a variety of drug discovery tasks (Figs. 2 and 3). In particular, we identified candidate anti-SARS-CoV-2 agents, which were validated by ongoing clinical and experimental data across 13 biological anti-SARS-CoV-2 assays. If broadly applied, our pre-training deep learning framework will offer a powerful tool for rapid drug discovery and development for various emerging diseases, including COVID-19 pandemic and future pandemics as well.

We highlighted several improvements of ImageMol compared to other state-of-the-art methods. First, ImageMol achieved high performance across diverse tasks of drug discovery, including drug-like property assessment (brain permeability, drug’s metabolism and toxicity) and molecular target prediction across diverse targets, such as Alzheimer’s disease (i.e., BACE) and emerging infectious diseases caused by HIV and SARS-CoV-2 virus. Furthermore, ImageMol outperforms state-of-the-art methods, including traditional deep learning and machine learning models (Fig. 2a–2c). Second, we showed that our self-supervised image-based representation approach outperformed traditional fingerprint-based and graph-based representation methods as well (Fig. 2d–2f). Finally, ImageMol has better interpretability and is more intuitive in identifying biologically relevant chemical structures or substructures for molecular properties and target binding (Figs. 4a–4c). Via ablation analysis, we showed that pre-training process using 8.5 million drug-like molecules significantly improved the model performance compared to models without pre-training. Thus, integrating additional chemical knowledge (such as atomic properties and 3D structural information) to each image or pixel area may further improve the performance of ImageMol. We found that five pre-training tasks are well compatible and jointly improve model performance.

We acknowledged several limitations in current study. Although we mitigated the effects of different representations of molecular images through data augmentation, perturbed views (i.e., rotation and scaling) of the input images may still affect the prediction results of ImageMol. We did not optimize for the sparsity of molecular images, which may affect the latent features extracted by the model. It is challenging to explicitly define the chemical properties of atoms and bonds compared to graph-based methods [23, 34], which will inevitably lead to insufficient chemical information. Several potential directions may improve our ImageMol further: (1) integration of larger-scale biomedical data and larger-capacity models (such as ViT [45]) in molecular images will inevitably be the focus of future work; (2) multi-view learning of joint images and other representations (e.g. SMILES and graph) is an important research direction; (3) introducing more chemical knowledge (including atomic properties, 3D information, etc.) to each image or pixel area is also a point worth studying as well. In summary, ImageMol is an active self-supervised image processing-based strategy that offers a powerful toolbox for computational drug discovery in a variety of human diseases, including COVID-19.

## Online Methods

### Strategies for pre-training ImageMol

Pre-training aims to make the model learn how to extract expressive representations by training on large-scale unlabeled datasets and then apply the well pre-trained model to related downstream tasks and fine-tune to improve their performance. Defining several effective and task related pretext tasks is required for pre-training the model. In this paper, the core of our pretraining strategy is the visual representation of molecules by considering three principles: consistency, relevance, and rationality. These principles lead ImageMol to capture meaningful chemical knowledge and structural information from molecular images. Especially, the consistency means that the semantic information of the same chemical structure in different images is consistent, such as −OH, =O, benzene. The relevance means that different augmentations of the same image (such as mask, shuffle) are related in the feature space. For example, the distribution of the image after the mask should be close to the original image. The rationality means that the molecular structure must conform to chemical common sense. The model needs to recognize the rationality of the molecule in order to promote the understanding of the molecular structure. Unlike graph-based and smiles-based pre-training methods (they either only consider consistency or only correlation), ImageMol is the first molecular image-based pre-training framework and considers multiple principles comprehensively by defined five effective pretext tasks.

### Consistency for pre-training

Considering that the semantic information of the same chemical structure in different images is consistent, the Multi-Granularity Chemical Clusters Classification (MG3C) task is proposed (Supplementary Figure 1), which discovers semantic consistency by predicting the chemical structure of the molecule. Briefly, multi-granularity clustering is first used to assign multiple clusters of different granularities to each chemical structural fingerprint. Then, each cluster is assigned as a pseudo-label to the corresponding molecule and each molecule has multiple pseudo-labels with different granularities; Finally, molecular encoder is employed to extract the latent features of the molecular images and a structural classifier is used to classify the pseudo-labels.

Especially, we employed the *MACCS* keys as the descriptor of molecular fingerprints, which is a 166-length sequence composed of 0 and 1. These molecular fingerprint sequences can be used as a basis for clustering, and the closer the distance between molecular fingerprints, the more likely it is to be clustered into a cluster. Finally, we use the *K-means* [46] with different *K=100,1000,10000* (See Supplementary Section A.2 and Supplementary Figure 15 about selection of *K*) to cluster molecules to obtain clusters with different granularity from coarse-grained to fine-grained. According to the clustering results, we assigned three pseudo-labels to each molecular image and then applied ResNet18 [47] as molecular encoder to extract latent feature and structural classifier to predict the pseudo-labels of latent feature. The structural classifier is multi-task, consisting of three parallel fully connected layers corresponding to three different clustering granularities. The neurons of each fully connected layer are 100, 1000 and 10000, respectively. Formally, the molecular image and the corresponding three pseudo-labels are represented by $x_{n}\in {\mathbb{R}}^{224\times 224\times 3}$, $Y_{n}^{100}\in {\left\{0,1,\ldots ,99\right\}}^{100}$, $Y_{n}^{1000}\in {\left\{0,1,\ldots ,999\right\}}^{1000}$ and $Y_{n}^{10000}\in {\left\{0,1,\ldots ,9999\right\}}^{10000}$, respectively and the cost function $L_{MG3C}$ of multi-granularity chemical clusters classification task is as follows:

(1.) $$L_{MG3C}=\underset{\theta ,W}{\text{arg}\;\text{min}}\frac{1}{N}\sum_{n=1}^{N}\ell \left(w_{100}\left(f_{\theta }\left(x_{n}\right)\right),Y_{n}^{100}\right)+\ell \left(w_{1000}\left(f_{\theta }\left(x_{n}\right)\right),Y_{n}^{1000}\right)+\ell \left(w_{10000}\left(f_{\theta }\left(x_{n}\right)\right),Y_{n}^{10000}\right)$$

Where *f*_*θ*_ and *θ* refer to the mapping function and corresponding parameters of molecular encoder, respectively. *w*_100_, *w*_1000_ and *w*_10000_ represent the parameters of three fully connected classification layers in structural classifier with 100, 1000 and 10000 neurons, respectively. *W* represents all parameters of *w*_100_, *w*_1000_ and *w*_10000_. *ℓ* is the multinomial logistic loss or the negative log-softmax function.

### Relevance for pre-training

Based on the assumption that different augmentations (such as mask, shuffle) of the same image are related in the feature space, we use a pixel-level task to reconstruct molecular images from latent features and use an image-level task to maximize the correlation between the original sample and the mask sample in that space.

#### Molecular image reconstruction (MIR).

MIR reconstruct the latent features back to the molecular images. We input the original molecular image *x*_*n*_ into molecular encoder to obtain the latent feature *f*_*θ*_(*x*_*n*_). To make the model learn the correlation between the molecular structures in the image, we shuffle and rearrange the input image *x*_*n*_ (as in Section **Rationality for pretraining**) in the hope that the correct image can be reconstructed. After that, we define a generator *G* and a discriminator *D* to reconstruct the latent features. *G* is composed of four layers of 2D deconvolution layers with a batch normalization 2D layer and ReLU activation function, and one layer of deconvolution layer with a Tanh activation function. The discriminator is also composed of four layers of 2D convolutional layers with a batch normalization 2D layer, a LeakyReLU activation function, and one layer of 2D convolutional layer with Sigmoid activation function. For further details of the GAN model see Supplementary Figure 5. Since it is difficult for the generator to reconstruct the latent features to 224 × 224 molecular images, we simplify the task to reconstruct the latent features to 64 × 64 molecular images. The discriminator accepts 64 × 64 molecular images and distinguishes real or fake images. In detail, first the generator is used to reconstruct the latent feature *f*_*θ*_(*x*_*n*_) to a 64 × 64 molecular image ${\tilde{x}}_{n}^{64\times 64}=G\left(f_{\theta }\left(x_{n}\right)\right)$. Then, we resize the original molecular image *x*_*n*_ of 224 × 224 to the molecular image $x_{n}^{64\times 64}$ of 64 × 64 and input it into the discriminator *D* together with the molecular image generated by *G* at the same time to obtain $D\left(x_{n}^{64\times 64}\right)$ and *D*(*G f*_*θ*_(*x*_*n*_)). Finally, we update the parameters of the generator and the discriminator through their cost functions $L_{G}\;$ and $L_{D}$ respectively, which are defined as:

(2.) $$L_{G}=E\left[D\left(G\left(f_{\theta }\left(x_{n}\right)\right)\right)\right]+{\left\|G\left(f_{\theta }\left(x_{n}\right)\right),x_{n}^{64\times 64}\right\|}_{2}$$

(3.) $$L_{D}=E\left[D\left(x_{n}^{64\times 64}\right)\right]-E\left[D\left(G\left(f_{\theta }\left(x_{n}\right)\right)\right)\right]$$

For $L_{G}$, the first term represents Wasserstein loss, and the second term represents the Euclidean distance between the generated image *G*(*f*_*θ*_(*x*_*n*_)) and the corresponding real image $x_{n}^{64\times 64}$. For $L_{D}$, we use this loss to approximate the Wasserstein distance of the distribution of real features $x_{n}^{64\times 64}$ and fake features *x*_*n*_. Finally, the molecular encoder model is updated by using the cost function $L_{MIR}$, which was formalized as

(4.) $$L_{MIR}=E\left[D\left(G\left(f_{\theta }\left(x_{n}\right)\right)\right)\right]+{\left\|G\left(f_{\theta }\left(x_{n}\right)\right),x_{n}^{64\times 64}\right\|}_{2}-E\left[D\left(G\left(f_{\theta }\left(x_{n}\right)\right)\right)\right]$$

#### MASK-based contrastive learning (MCL).

Recently, the performance gap between the unsupervised pre-training and supervised leaning in computer vision has narrowed, notably owing to the achievements of contrastive learning methods [25, 26]. However, these methods typically rely on a large number of explicit pairwise feature comparisons, which is computationally challenging [48]. Furthermore, in order to maximize the feature extraction ability of the pre-training model, contrastive learning must select good feature pairs, which obviously increases the huge cost in computing resources. Therefore, to save computing resources and mine the fine-grained information in the molecule images, we introduce a simple contrastive learning method in molecular images, namely MASK-based contrastive learning (Supplementary Figure 4). We first use a 16 × 16 square area to randomly mask the molecular images (Supplementary Figure 16), denoted by ${\tilde{x}}_{n}$. Then, the masked molecular images ${\tilde{x}}_{n}$ and the unmasked molecular images *x*_*n*_ are simultaneously input into molecular encoder to extract latent features $f_{\theta }\left({\tilde{x}}_{n}\right)$, *f*_*θ*_(*x*_*n*_). Finally, the cost function $L_{MCL}$ was introduced to ensure the consistency between the latent feature extracted by the molecular image before and after the mask, which was formalized as:

(5) $$L_{MCL}=\underset{\theta }{\text{arg}\;\text{min}}\frac{1}{N}\sum_{n=1}^{N}{\left\|f_{\theta }\left({\tilde{x}}_{n}\right),f_{\theta }\left(x_{n}\right)\right\|}_{2}$$

Where ${\left\|f_{\theta }\left({\tilde{x}}_{n}\right),f_{\theta }\left(x_{n}\right)\right\|}_{2}$ means to calculate the Euclidean distance between $f_{\theta }\left({\tilde{x}}_{n}\right)$ and *f*_*θ*_(*x*_*n*_).

### Rationality for pre-training

Inspired by human understanding of the world, we proposed the rationality principle, which means that the structural information described by molecular images must conform to chemical common sense. We rearranged the original images to construct irrational molecular images and designed two pre-training tasks to predict them (Supplementary Figure 2 and Supplementary Figure 3), which can effectively improve the model’s understanding of molecular images.

#### Molecular rationality discrimination (MRD).

The reason why people can easily judge whether things in the image are reasonable based on the knowledge they have learned is because people are very good at summarizing the spatial structure information in the image scene. For example, image of a blue sky under the grass and an image of a blue sky above the grass, we can easily distinguish the former is unreasonable and the latter is reasonable. However, it is difficult for an artificial intelligence model to pay attention to this global-level spatial structure information spontaneously during the learning process. Motivated by these phenomena, we construct a rational and an irrational molecular image pair for each molecular image to guide the model to learn the structural information. Especially, as shown in Supplementary Figure 2, we use an 3 × 3 grid to decompose each molecular image *x*_*n*_ into 9 patches and number each patch 1 to 9. Then, these patch numbers are randomly shuffled and re-spliced according to the shuffled patch to form an image with the same dimensions as the original image. Finally, these disordered images are viewed as irrational samples ${\hat{x}}_{n}$. Subsequently, the original ordered image *x*_*n*_ and the shuffled image ${\hat{x}}_{n}$ are forward propagated to molecular encoder to extract latent features *f*_*θ*_(*x*_*n*_) and $f_{\theta }\left({\hat{x}}_{n}\right)$, and these features are further input into a rationality classifier to obtain the probability value *w*_*MRD*_*f*_*θ*_(*x*_*n*_) whether the sample is reasonable. Here, we define the cost function of molecular rationality discrimination task $L_{MRD}$ to update ResNet18, which is formalized as:

(6) $$L_{MRD}=\underset{\theta ,w_{MRD}}{\text{arg}\;\text{min}}\frac{1}{N}\sum_{n=1}^{N}\ell \left(w_{MRD}\left(f_{\theta }\left(x_{n}\right)\right),Y_{n}^{MRD}\right)+\ell \left(w_{MRD}\left(f_{\theta }\left({\hat{x}}_{n}\right)\right),Y_{n}^{MRD}\right)$$

Where the first term and the second term represent the binary classification loss of the rational image and the irrational image respectively. *w*_*MRD*_ represents the parameters of the rationality classifier. $Y_{n}^{MRD}$ represents the real label, which consists of 0 (irrational) and 1 (rational).

#### Jigsaw puzzle prediction (JPP).

Compared with MRD, JPP provides a more fine-grained prediction to discover the invariance and regularity of molecular images (Supplementary Figure 3), which is widely used in computer vision [49]. Solving a jigsaw puzzle on the same molecular images can help the model pay attention to the more global structural information and learn the concepts of spatial rationality to improve the generalization of the pre-training model. In this task, by using the maximal Hamming distance algorithm in [50], we assign an index (ranging from 0 to 99) to each permutation of patch numbers, which will be used as the classification label $Y_{n}^{Jig}$ of the molecular image. Similar to MRD task, the original ordered image *x*_*n*_ and the shuffled image ${\hat{x}}_{n}$ are forward propagated to molecular encoder to extract latent features *f*_*θ*_(*x*_*n*_) and $f_{\theta }\left({\hat{x}}_{n}\right)$. Then, an additional jigsaw classifier is introduced to classify the permutation to which the image belongs. The molecular encoder is updated by using cost function $L_{JPP}$, which is formalized as:

(7) $$L_{JPP}=\underset{\theta ,w_{Jig}}{\text{arg}\;\text{min}}\frac{1}{N}\sum_{n=1}^{N}\ell \left(w_{Jig}\left(f_{\theta }\left(x_{n}\right)\right),Y_{n}^{Jig}\right)+\ell \left(w_{Jig}\left(f_{\theta }\left({\hat{x}}_{n}\right)\right),Y_{n}^{Jig}\right)$$

Where the first term and the second term represent the classification loss of the original ordered image and the shuffled image respectively. *w*_*jig*_ represents the parameters of the jigsaw classifier.

### Pre-training process

In pre-training, we used two large-scale datasets (ZINC and ChEMBL) for unsupervised pre-training. ZINC is a dataset containing 8 million unlabeled molecules sampled from the ZINC15 database, and ChEMBL is a smaller dataset containing ~0.43 million unlabeled molecules. The two datasets have been preprocessed and are publicly available online [23]. Overall, the pretraining of ImageMol consists of two steps, which are data augmentations and training process, respectively. A detailed pre-training data flow can be found in Supplementary Figure 17 and Supplementary Section B.2.

#### Data augmentations.

Data augmentation is a simple way to effectively augment a limited number of samples and significantly improve the generalization ability and robustness of the model, which has been widely used in supervised and unsupervised representation learning. However, different from ordinary images, the molecular images are relatively sparser as they are filled mostly (>90%) by zeros, resulting in “usable” data being limited to a very small fraction of the image [29]. In view of the above limitation, “random cropping” is not applied in our model. Finally, three augmentations are selected in pre-training stage, including *RandomHorizontalFlip*, *RandomGrayscale* and *RandomRotation*. Hence, before the original images are input into our pre-training model, each image has a 50% probability of being horizontal flipped, 20% probability of being converted to grayscale, and 100% probability of being rotated between 0°–360°. The augmentations are provided by PyTorch (https://pytorch.org/).

#### Training process.

Here, we used the ResNet18 as our molecular encoder. After using data augmentations to obtain molecular images *x*_*n*_, we forward these molecular images *x*_*n*_ to the ResNet18 model to extract latent features *f*_*θ*_(*x*_*n*_). Then, these latent features are used by five pretext tasks to calculate the total cost function $L_{ALL}$, which is defined as:

$$L_{ALL}=L_{MG3C}+L_{JPP}+L_{MIR}+L_{MRD}+L_{MCL}$$

Finally, the total loss function $L_{ALL}$ is used for backpropagation to update ResNet18. Specially, the cost function $L_{ALL}$ is minimized using mini-batch stochastic gradient descent (SGD). See Supplementary Section A.3 and Supplementary Table 14 for more detailed hyperparameter settings and Supplementary Section C.1 and Supplementary Figure 18 for the loss record during pre-training.

### Fine-tuning

After completing the pre-training, we fine-tune the pre-trained ResNet18 in the downstream task. Clearly, the performance of the model can be further improved by establishing a complex fine-tuning task for the pre-trained model. However, fine-tuning is not the research focus of this paper, so we only use a simple and common fine-tuning method to adapt the model to different downstream tasks. In detail, we only add an additional full connection layer *g*^*ft*^ after the ResNet18, and the output dimension of the full connection layer is equal to the number of classifications of downstream tasks. In fine-tuning, we first input the molecular image $x_{n}^{gt}$ from the downstream task into ResNet18 to obtain the latent feature representation $f_{\theta }\left(x_{n}^{gt}\right)$. Then, we forward the latent feature representation to the full connection layer *g*^*ft*^ to obtain the logical value $g^{ft}\left(f_{\theta }\left(x_{n}^{gt}\right)\right)$ related to the category and use the softmax activation function to normalize these logical values to get the predicted category probability ${\tilde{Y}}_{n}^{gt}=\text{softmax}\left(g^{ft}\left(f_{\theta }\left(x_{n}^{gt}\right)\right)\right)$. Finally, our model will be fine-tuned by calculating the cross-entropy loss between the category probability ${\tilde{Y}}_{n}^{gt}$ and the true label $Y_{n}^{gt}$. Especially, since the data in the downstream task has the problem of category imbalance, we also added the category weight in the cross-entropy loss, which is formalized as:

(8) $$L_{CE}=-\frac{1}{N}\left[\sum_{i=1}^{N}\sum_{k=1}^{K}\lambda_{k}Y_{i,k}^{gt}\text{log}{\tilde{Y}}_{i,k}^{gt}\right]$$

Where, *N* and *K* respectively represent the number of samples and the number of categories in downstream tasks. *λ*_*k*_ represents category weight, which is calculated by $1-\frac{N_{k}}{N}$ (*N*_*k*_ is the number of samples of category *k*). $Y_{i,k}^{gt}$ and ${\tilde{Y}}_{i,k}^{gt}\;$ represent the true label and predicted probability on the *k*-th category of the *i*-th sample. Finally, the loss function $L_{CE}$ is used for backpropagation to update the parameters of the model. The more detailed hyperparameter settings can be found in Supplementary Section A.3 and Supplementary Table 14.

### Downstream task details

To evaluate our proposed pre-training model, we designed three types of downstream tasks related to molecular representation learning for testing, which are molecular property prediction, drug metabolism prediction and antiviral activities prediction, respectively.

### Molecular property prediction

#### Dataset.

MoleculeNet [51] is a popular benchmark for molecular property prediction. Here, we used five binary classification datasets (Tox21, ClinTox, BBBP, HIV, and BACE) from MoleculeNet to evaluate our ImageMol. See Supplementary Table 1 for details. In these five datasets, Tox21 is complex multiple binary classification tasks, with 12 tasks and 7831 samples. ClinTox has two binary classification tasks and 1478 samples. The three remaining classification datasets (BBBP, HIV, and BACE) are single binary classification tasks with 2039, 41127, 1513 samples respectively.

#### Comparison method.

For a comprehensive comparison, we selected several different types of popular methods, which are the SMILES sequence-based pre-training methods (ChemBERTa [21], SMILES Transformer [22], RNNS2S [32] and Mol2Vec [33]), the graph-based pre-training methods (Jure’s GNN [23] and N-GRAM [34]) and molecular image-based method (Chemception [29]). These recently proposed methods show competitive results and superior performance on molecular property prediction task. Therefore, we selected these representative methods for comparison. In the sequence-based pre-training methods, ChemBERTa is based on RoBERTa [52] with 12 attention heads, 6 layers, and pre-trained by 77M unique SMILES sequences from PubChem [53]; the SMILES Transformer builds an encoder-decoder network with 4 transformer [54] blocks, which is pretrained with 861,000 unlabeled SMILES sequences randomly sampled from ChEMBL24 [27]; the RNNS2S is designed based on sequence-to-sequence learning with GRU [55] cell and attention mechanism, which is pretrained by using 334,092 valid molecular SMILES sequences from LogP and PM2-full datasets. Mol2Vec learns vector representations of molecular substructures that point in similar directions for chemically related substructures by pre-training on 19.9 million compounds. In the graph-based pre-training method, Jure’s GNN are node-level cutting edge self-supervised pre-training methods, which first transform the 2M SMILES sequences sampled from the ZINC15 database [28] into a graph structure, and use different pre-training strategies to train the Graph Isomorphism Networks (GINs) [56]. The N-GRAM method introduces N-gram graph and learns a compact representation for each graph in pretraining. Within molecular image-based methods, Chemception [29] has a well-designed CNN architecture focused on molecular property prediction. To quantitatively compare the advantages and disadvantages of ImageMol and these methods, ROC–AUC score is calculated as the evaluation metric.

#### Experimental setting.

Due to the differences in data split between different methods, for fair comparison, we used multiple different data split ways to comprehensively evaluate our ImageMol. In order to compare fairly with RNNS2S [32] and SMILES Transformer [22], we split the original dataset into training set (80%), validation set (10%) and test set (10%) with stratified split. At the same time, to evaluate the stability of our results, we use different random seeds to perform 20 times and take the mean and variance of these results as the final result. Compared to a stratified split, the scaffold split is a more challenging and realistic evaluation setting because molecular substructures do not overlap between training and test sets. Therefore, we follow the experimental setup of Jure’s GNN [23] to use scaffold split to divide experimental datasets into training set (80%), validation set (10%) and test set (10%). The final performance will be reported by calculating the mean and variance of the experimental results from 5 different random seeds. In addition, in order to compare with Chemception [29], we use exactly the same experimental configuration as Chemception, which uses stratified split to divide 4/6 training set, 1/6 validation set and 1/6 test set.

### Drug metabolism prediction

#### Dataset.

In drug discovery, Cytochrome P450 inhibitors and noninhibitors classification is important for predicting the tendency of molecules to cause significant drug interactions by inhibiting CYP and to determine which subtypes are affected. In this task, we use PubChem Data Set I (Training Set) and PubChem Data Set II (Validation Set) from [35] to evaluate the performance of the proposed ImageMol on human cytochrome P450 (CYP) inhibition. PubChem Data Sets I and II are two-category datasets. Both include 1A2, 2C9, 2C19, 2D6 and 3A4 isoforms.

#### Comparison method.

We compare the proposed ImageMol with two latest molecular image-based methods (ADMET-CNN [30] and QSAR-CNN [31]) with ROC-AUC metric to confirm the superiority of our method on molecular images and other molecular fingerprinting-based methods (MACCS-based and FP4-based methods [35]) with accuracy and ROC-AUC metrics to validate that our method can learn more information from molecular images than molecular fingerprints. For molecular image-based methods, ADMET-CNN successfully established a molecular 2-D image-based CNN model and achieved good prediction performances on predicting the ADMET properties (including CYP1A2 inhibitory potency, P-gp inhibitory activity, etc.); QSAR-CNN applied transfer learning and data augmentation to train molecular image-based DenseNet121 [57] model for developing quantitative structure-activity relationships (QSARs) to predict compound rate constants toward OH radicals. For molecular fingerprinting-based methods, two types of methods are used in the comparison, which includes traditional machine learning methods (SVM, C4.5 DT, *k*-NN and NB) and ensemble learning methods (CC-I, CC-II, etc.) respectively. In this task, the accuracy and ROC-AUC are calculated for comparison.

#### Experimental setting.

For fairness, we keep the experimental settings consistent with these methods. We use 5-fold cross-validation on PubChem Data Set I to evaluate the performance of our ImageMol, ADMET-CNN and QSAR-CNN. In addition, we also use the model trained in PubChem Data Set I to evaluate the performance of all models mentioned in this task on the external validation set PubChem Data Set II.

### Anti-SARS-CoV-2 activities prediction

#### Dataset.

Anti-viral activities prediction is vital for the development of new drugs to treat COVID-19. We then use anti-SARS-CoV-2 activities prediction as our task to prioritize compounds when screening in vitro. The experimental datasets are obtained from the COVID-19 portal [20] in the National Center for Advancing Translational Sciences (NCATS), which include 13 assays such as Spike-ACE2 protein-protein interaction (AlphaLISA), Spike-ACE2 protein-protein interaction (TruHit Counterscreen), ACE2 enzymatic activity, etc. These 13 assays represent five distinct categories: viral entry, viral replication, live virus infectivity, counterscreen and in vitro infectivity. Due to the extreme imbalance in these original datasets, the proportion of positive samples in the total samples ranges from 0.7% to 7.3%, so we filter out those samples without AC_50_ to generate our datasets and set AC_50_ greater than 10 and less than 10 as non-inhibitors and inhibitors, respectively. The overview of the processed datasets is summarized in Supplementary Table 6.

#### Comparison method.

We chose two representative methods for experimental comparison, Jure’GNN [23] and REDIAL-2020 [20]. Jure’GNN is a pre-training method based on graph and graph neural network (GNN), which used molecular graph as the input data of the GNN and introduced a series of pre-training strategies to train the GNN to obtain better molecular embedding. REDIAL-2020 is a suite of computational models based on manual features, which extracts a total of 22 features of three different types (19 fingerprints-based, 1 pharmacophore-based and 2 physicochemical descriptors-based) to train the machine learning model from scikit-learn package. In this task, we used a total of 6 evaluation metrics, namely accuracy, sensitivity, precision, ROC-AUC, AUPR and F1.

#### Experimental setting.

In order to compare our ImageMol with Jure’s GNN, we reproduced Jure’s GNN by using the public source code they provided to extract molecular features and added a fully connected layer for fine-tuning on downstream tasks. We uniformly split these datasets into 80% training set and 20% test set, and report the AUC and AUPR results on test set. We also compared our method with REDIAL-2020. To compare fairly with REDIAL-2020, we use the same experimental configuration as REDIAL-2020. See [20] for detailed experimental setting. Note that REDIAL-2020 provides a new data preprocessing method and divides the training set, validation set and test set, so we directly use these divided datasets to perform our evaluation process (Supplementary Table 13). For the experimental results, we use the model that achieves the best performance on the validation set to evaluate the results of the test set. Finally, accuracy, F1, sensitivity, precision and ROC-AUC metrics are reported in the experiment.

## Supplementary Material

Supplement 1

Supplement 2

## Figures and Tables

**Figure 1.** A diagram illustrating the ImageMol framework. (**a**) A molecular encoder (light blue) is used to extract the latent features of the molecular images. (**b**) The five strategies are used to pretrain the molecular encoder. The structural classifier (dark blue) in multi-granularity chemical clusters classification (MG3C) is used to predict chemical structural information in molecular images. The rationality classifier (green) in molecular rationality discrimination (MRD) is used to distinguish rational and irrational molecules. The jigsaw classifier (grey) in jigsaw puzzle prediction (JPP) is used to predict rational permutations. The contrastive classifier (orange) in MASK-based contrastive learning (MCL) is used to maximize the similarity between the original image and the masked image. The generator (yellow) in molecular image reconstruction (MIR) is used to restore latent features back to the molecular image and the discriminator (purple) is used to discriminate between real and fake molecular images. (**c**) ImageMol for discovery of anti-SARS-CoV-2 inhibitors. A fully connected (FC) layer is appended to the pretrained molecular encoder for fine-tuning on the COVID-19 dataset. Subsequently, the fine-tuned model is used for virtual screening from approved drugs in the DrugBank. The 65% success rate of the top 20 drugs have been validated by experimental and clinical evidence as potential inhibitors of COVID-19.

**Figure 2.** Performance evaluation of ImageMol using the benchmark datasets. The performance was evaluated in a variety of drug discovery tasks, including molecular properties (i.e., drug’s metabolism, toxicity, brain penetration) and molecular target profiles (i.e., human immunodeficiency virus (HIV) and beta-secretase (BACE)). The x-axis and y-axis represent False Positive Rate (FPR) and True Positive Rate (TPR) in **a**-**c**, respectively. (**a**) Receiver operating characteristic (ROC) curves of ImageMol across five datasets (blood-brain barrier penetration (BBBP), molecular Toxicity using the 21st century (Tox21), clinical trial toxicity (ClinTox), HIV and BACE) with stratified split and scaffold split. (**b**) ROC curves of Chemception [29] and ImageMol on Tox21 and HIV datasets with the same experimental setup as Chemception, which is a classical convolutional neural network (CNN) for predicting molecular images. (**c**) ROC curves of ADMET-CNN [30], QSAR-CNN [31] and ImageMol on five CYP isoforms validation sets (PubChem Data Set II). ADMET-CNN and QSAR-CNN are the latest molecular image-based drug discovery models. (**d**) The ROC-AUC (%) performance of SMILES-based methods (SMILES Transformer [22], Recurrent Neural Network-based Sequence-to-Sequence (RNNS2S) [32]) and ImageMol on four datasets (BBBP, Tox21, HIV and BACE) with stratified split. (**e**) The ROC-AUC (%) performance of SMILES-based, graph-based methods and ImageMol on four MPP datasets (BBBP, ClinTox, HIV and BACE) with scaffold split. (**f**) The ROC-AUC (%) performance of fingerprint-based (MACCS-based and FP4-based) methods and ImageMol across five major Cytochrome P450 (CYP) Isoforms.

**Figure 3.** Evaluation and Discovery of anti-SARS-CoV-2 inhibitors using ImageMol. (**a**) Receiver operating characteristic (ROC) curves of Jure’s GNN and ImageMol across 13 high-throughput experimental SARS-CoV-2-related datasets. The x-axis and y-axis represent False Positive Rate (FPR) and True Positive Rate (TPR), respectively. (**b**) t-SNE visualization on molecular images with activity concentration 50% (AC_50_) by using the latent features of the global average pooling (GAP) layer of our ImageMol. Red means active and blue means in-active molecules. (**c**) t-SNE visualization of the 3CL dataset from anti-viral activity prediction task. The molecules with AC_50_ less than 10 were treated as inhibitors and greater than 10 were treated as non-inhibitors. From red to blue indicates the probability distribution of 3CL non-inhibitors and inhibitors. (**d**) Drug discovery of 3CL potential inhibitors on DrugBank dataset. The black dots represent the probability distribution of drug molecules from DrugBank, and the white dots represent the known 3CL inhibitors found by ImageMol. (**e**) Molecular structure of the 3CL inhibitor discovered by ImageMol.

**Figure 4.** Biological Interpretation of ImageMol. **(a)** Examples of otherwise the opposite is true. (**b** and **c**) The ImageMol’s heatmaps of several molecular images whose structures are highlighted by Gradient-weighted Class Activation Mapping (Grad-CAM). The warmer the color, the higher the attention of the area, and the colder the color, and the lower the attention of the area. In particular, the red area indicates that the model has the highest attention to it, while the light blue indicates that the model does not have any attention to it. (**d**) The average heat map of all molecular images on each dataset, which uses Grad-CAM to obtain the heat map of each molecular image in dataset and calculate the average of these heat maps in each dimension. (**e**) The variable probability distribution figures (principal diagonal) and the kernel density estimate figures (sub-diagonal) of representations learned by ImageMol. The representations extracted by ImageMol are dimensionally reduced by t-SNE. The different colors indicate different clusters.

## Data Availability

The datasets used in this project can be found at the following links: blood-brain barrier penetration (BBBP): http://deepchem.io.s3-website-us-west-1.amazonaws.com/datasets/BBBP.csv, beta-secretase (BACE): http://deepchem.io.s3-website-us-west-1.amazonaws.com/datasets/bace.csv, human immunodeficiency virus (HIV): http://deepchem.io.s3-website-us-west1.amazonaws.com/datasets/hiv.csv, molecular Toxicity using the 21st century (Tox21): http://deepchem.io.s3-website-us-west-1.amazonaws.com/datasets/tox21.csv.gz, clinical trial toxicity (ClinTox): http://deepchem.io.s3-website-us-west-1.amazonaws.com/datasets/clintox.csv.gz, 13 SARS-CoV-2 targets: https://opendata.ncats.nih.gov/covid19/assays (The corresponding dataset can be found in Supplementary Table 6), 5 drug’s metabolism enzymes: https://pubs.acs.org/doi/abs/10.1021/ci200028n, approved drug in DrugBank: https://go.drugbank.com/releases/5-1-9/downloads/approved-drug-links, 122 drugs that block SARS-CoV-2: https://static-content.springer.com/esm/art%3A10.1038%2Fs41586-022-04482-x/MediaObjects/41586_2022_4482_MOESM1_ESM.pdf.
