# Supplementary material for: Accurate prediction of molecular targets using a self-supervised image representation learning framework
Source: Res Sq. 2022 Apr 7:rs.3.rs-1477870. Preprint. [Version 1] doi: 10.21203/rs.3.rs-1477870/v1 (PMC8996628; doi:10.21203/rs.3.rs-1477870/v1)
Supplement: Supplement 2 [file 0a334b30a89c69deed6cda64.pdf]

Supplementary Materials

Accurate prediction of molecular targets using a self-supervised image representation learning framework

Xiangxiang Zeng[1,#], Hongxin Xiang[1,#], Linhui Yu[1], Jianmin Wang[1], Kenli Li[1], Ruth Nussinov[2,3], Feixiong Cheng[4-6,*]

[1]College of Computer Science and Electronic Engineering, Hunan University, Changsha, Hunan, 410082, China.

[2]Computational Structural Biology Section, Frederick National Laboratory for Cancer Research in the Laboratory of Cancer Immunometabolism, National Cancer Institute, Frederick, MD 21702, USA

[3]Department of Human Molecular Genetics and Biochemistry, Sackler School of Medicine, Tel Aviv University, Tel Aviv 69978, Israel

[4]Genomic Medicine Institute, Lerner Research Institute, Cleveland Clinic, Cleveland, OH 44195, USA

[5]Department of Molecular Medicine, Cleveland Clinic Lerner College of Medicine, Case Western Reserve University, Cleveland, OH 44195, USA

[6]Case Comprehensive Cancer Center, Case Western Reserve University School of Medicine, Cleveland, OH 44106, USA

*To whom correspondence should be addressed: Feixiong Cheng, Ph.D.

Lerner Research Institute, Cleveland Clinic, Ohio, USA

Tel: +1-216-444-7654; Fax: +1-216-636-0009

Email: chengf@ccf.org

Table of Contents

Supplemental Materials 3
A. Experiment setup 3
A.1 Datasets 3
A.2 Selection of K in *K-means* 4
A.3 Hyperparameters of pre-training and finetuning 5
B. Methods 6
B.1 Molecular image and fingerprint generation 6
B.2 Pre-task details in pre-training 6
C. Results 10
C.1 Results on the pre-training 10
C.2 Performance comparison with existing approaches 10
C.3 Results on anti-viral activities across SARS-CoV-2 targets 13
C.4 Results on the virtual screening anti-SARS-CoV-2 drugs 13
Supplementary Figures 15
Supplementary Tables 32
Supplementary References 44

Supplemental Materials

A. Experiment setup

A.1 Datasets

We used three different types of datasets for molecular property prediction, drug metabolism prediction and anti-viral activity prediction tasks.

Datasets of Molecular Property Prediction

The statistical information of MPP datasets is described in Table **S1**, which include BBBP, Tox21, HIV, ClinTox and BACE datasets:

- **BBBP** (Blood-Brain Barrier Penetration) dataset includes binary-classification records of barrier permeability properties between blood and brain of more than 2000 compounds.
- **Tox21** (Toxicology in the 21st Century) is a dataset of compound toxicity, including qualitative toxicity measurements for 8k compounds on 12 different targets.
- **HIV** (Human Immunodeficiency Virus) dataset contains more than 40,000 records of whether the compound inhibits HIV replication for binary classification between active and inactive.
- **ClinTox** (Clinical trial Toxicity) dataset includes 1491 drug compounds with known chemical structures for the binary classification between clinical trial toxicity (or absence of toxicity) and FDA approval status.
- **BACE** (BetA-seCretasE) dataset contains compounds that can be

inhibitors of human β-secretase 1 (BACE-1).

Datasets of Drug Metabolism Prediction

The PubChem Data Set I (Training Set) and PubChem Data Set II (Validation Set) are used as DMP (Drug Metabolism Prediction) datasets, which include binary-classification records of whether the compounds are the Cytochrome P450 inhibitors. The five major CYP isoforms in cytochrome P450 are used, namely 1A2, 2C9, 2C19, 2D6, and 3A4. The statistical information is described in Table **S2**.

Datasets of Anti-Viral Activity Prediction

The statistical information of the processed datasets is shown in Table **S6**. Each dataset contains binary-classification records of whether to inhibit SARS-CoV-2 activity. The records in each dataset only contain the inhibitory effects of compounds on a certain enzyme or a certain intermediate process, and the enzyme or other intermediate process will directly affect the expression of SARS-CoV-2 instead of directly acting on the SARS-CoV-2 through these compounds.

In addition, for a fair comparison with other method, we also used the SARS-CoV-2 datasets in [1] to train some models of anti-SARS-CoV-2 activities, and its statistical information is shown in Table **S13**.

A.2 Selection of K in *K-means*

In the clustering pseudo-label classification task, we determined the K

values to be 100, 1000, and 10000, respectively. In order to determine the value of K in K-Means method, we first use different K values, ranging from 1 to 14000, to cluster the dataset and to calculate the sum of squared distances. Then, we use the K value as the x-axis and sum of squared distances as the y-axis to draw a curve. Finally, a knee point detection algorithm [2] is used to find the knee point of this curve. As shown in Figure **S15**, the dotted line indicates the K value corresponding to the "elbow" point. Obviously, the larger the K value, the more difficult it is for ImageMol to perform the clustering pseudo-label classification task. Therefore, we select two K values (K =100 and 1000) on the left side of the "elbow" point and one K value (k=10,000) on the right side of the "elbow" point.

A.3 Hyperparameters of pre-training and finetuning

The hyperparameters of the pre-training and fine-tuning process are shown in Table **S14**. In the pre-training task, our model is pre-trained by SGD optimizer with learning rate 0.01, weight decay 10^{-5}, momentum 0.9 and batch size 256 for approximately 6 days on the Amazon server of the instance p3.16xlarge with 8 Tesla V100 GPU (32G). In downstream task, the pre-trained model is fine-tuned using SGD optimizer with batch size [8, 16, 32, 64, 128], learning rate [5e-5, 0.005, 0.01], weight decay 10^{-5}, momentum 0.9 and batch size 128 on Ubuntu 18.04.1 with Intel(R) Xeon(R) Platinum 8259CL CPU @ 2.50GHz and Tesla T4 (16GB).

B. Methods

B.1 Molecular image and fingerprint generation

In this study, we use image as molecular representation. To obtain molecular images, the RDKit (https://github.com/rdkit/rdkit) is used to generate standard and unique image [3] for each molecule. Unlike molecular graph, molecular image is composed of a pile of pixels rather than vertices and edges. In detail, we first filter out molecules without SMILES information in the original dataset. Second, we transform the SMILES sequences to molecular images using RDKit and set the image size to 224 × 224. Finally, these molecular images with the same size will be used as the initial dataset of our method. Considering that molecular fingerprints are easy to obtain and can express some priori knowledge of molecules, we chose *MACCS keys* to assist our pre-training process to make our model learn molecule-related priori knowledge. The *MACCS keys* are one of the commonly used structural molecular fingerprint [4], which contain 166 keys related to molecular structure. In our work, we used RDKit to generate a distinct 166-D molecular fingerprint for each molecule.

B.2 Pre-task details in pre-training

This section will describe the pre-training details of ImageMol with five pre-tasks. The overall data flow of the ImageMol framework during training is shown

in Figure **S17**. In general, the original input images X is processed into three different datasets. Augmented images X^{aug} is obtained by using data augmentation on X, including *RandomHorizontalFlip()*, *RandomGrayscale(p=0.2)* and *RandomRotation(degrees=360)* in torchvision. Shuffled images X^{Jig} is obtained by performing a jigsaw puzzle on X^{aug}. The puzzle rule uses "permutations 100" in [5]. Masked images X^{mask} is obtained by adding the mask matrix in X^{aug}, and the values in the matrix are filled with the mean value. The examples about masked images are shown is Figure **S16**. Then, randomly select a batch of data from these three datasets and input them into ResNet18 without classification layer to extract 512-D latent features $z^{aug}, z^{jig}, z^{mask}$. Finally, these latent features are input into the sub-network for each task for further processing.

Specially, Figures **S1-S5** show the architecture of each pre-training strategy. In multi-granularity chemical clusters classification (MG3C) task (Figure **S1**), chemical fingerprints are first extracted from SMILES and input into unsupervised KMEANS with different K values to produce clusters with different structure granularity. Then, these clusters are treated as pseudo-labels of molecular images. Finally, the molecular encoder and structural classifier are jointly used to predict the labels of molecular images and optimizing the loss between pseudo-labels and predicted labels in pre-training. The structural classifier is a multi-task learner that receives 512-dimensional features as input and then forward-propagates to 3 fully connected layers with different numbers

of neurons (100 , 1000 and 10000) for classifying different clustering granularity.

In molecular rationality discrimination (MRD) task (Figure **S2**), we first disrupt the molecular structure to construct an irrational molecular image, which uses a 3x3 grid to decompose the molecular image into 9 patches and randomly shuffle them to form an irrational image. The original images are viewed as rational molecular images. Then, these rational and irrational molecules will be input to the molecular encoder to extract 512-D features. Finally, these features are forward propagated to rationality classifier for rationality judgment. The rationality classifier is a simple MLP structure that takes 512-dimensional feature as input and directly outputs 2-dimensional results (rational or irrational).

In jigsaw puzzle prediction (JPP) task (Figure **S3**), similar to MRD, we first decompose the molecular image into 9 patches and label the original permutation as $(1,2,3,4,5,6,7,8,9)$. Then, we randomly shuffle the permutation and re-stitch into new images like $(7,1,6,2,0,5,4,3,8)$ or $(7,8,5,6,3,2,0,1,4)$. In particular, we randomly select from 100 defined permutations, which can be obtained from permutations_100.npy (https://github.com/fmcarlucci/JigenDG/blob/master/permutations_100.npy). Finally, the Molecular encoder is used to extract features of rearranged images and subsequently input into the jigsaw classifier for predicting the permutation (100 classification). The Jigsaw classifier is a simple MLP, which consists of a

512-dimensional input layer and a 100-dimensional output layer.

In MASK-based contrastive learning (MCL) task (Figure **S4**), we randomly mask a 16×16 region in the molecular image, which is filled using the mean of the image (some masked examples in Figure **S16**). Subsequently, image pairs (original image, masked image) are fed into the molecular encoder to extract features and maximize the similarity. Here, the Euclidean distance is used to constrain the similarity between two features, and we should minimize the Euclidean distance to ensure greater similarity.

In molecular image reconstruction (MIR) task (Figure **S5**), we build our GAN model based on context encoders [6]. The detail of GAN model is described in Figure **S5**. In generator, firstly, the latent features z^{aug} are forward to a single hidden layer MLP model, which accepts 512-d input and obtains a 128-d output. Subsequently, four ConvTranspose2D layer with BatchNorm2D and ReLU are used. In ConvTranspose2D, the numbers represent input channels, output channels, kernel size and stride respectively. Finally, a ConvTranspose2D layer with Tanh activation function is used to generate 64×64 images. In discriminator, X^{aug} is first preprocessed to resize to 64×64. Then resized X^{aug} and X^{rec} are input to a Conv2d with LeakyReLU and three Conv2d with BatchNorm2D and LeakyReLU (negative slope is 0.2). In Conv2d, the numbers have the same meaning as ConvTranspose2D. Finally, a Conv2d is used to discriminate the real or fake of input images.

C. Supplementary Results and Discussion

C.1 Results on the pre-training

As shown in Figure **S18**, it shows the details of the loss change of ImageMol during pre-training. We did not show the training details of the Image reconstruction task because the loss is adversarial. In general, the loss of ImageMol in the remaining four pre-tasks is a decreasing trend and gradually converges, which shows that our ImageMol can learn different information about molecular images in these pre-tasks.

C.2 Performance comparison with existing approaches

We compare ImageMol with four different types of models, which are fingerprint-based models, sequence-based models, graph-based models and image-based models, respectively.

Fingerprint-based models. We selected several state-of-the-art fingerprint-based methods [1, 7] to compare on drug metabolism benchmark, which are traditional models and their ensemble models based on MACCS and FP4 [7]. Specially, these traditional machine learning models include SVM, C4.5 Decision Tree (DT), k-Nearest Neighbors (KNN) and Naive Bayes (NB) and the ensemble models includes five different combinations of SVM, C4.5 (DT), KNN, NB and three ensemble strategies (Mean, Maximum, Multiply). We found that ImageMol can outperform these methods on almost all benchmarks (Figure **2.f** and Table **S3**), except for the accuracy metric on

CYP2C9 Isoform, which is only 0.8% lower, and the others are improved by 1.0%~4.4%, which shows the features extracted by ImageMol are richer than manual features.

Sequence-based models. Due to the simplicity and efficiency of the Simplified Molecular-Input Line-entry System (SMILES) sequence, it has become one of the most popular molecular representation [8, 9]. We compared our ImageMol with several popular pre-training models (RNNS2S [10], SMILES Transformer [11] and ChemBERTa [12], MolVec [13], N-GRAM [14]) in molecular targets, blood-brain barrier penetration, drug toxicity benchmarks. The performance of our ImageMol can outperform the state-of-the-art sequence-based pre-training models on any datasets (Figure **2.d** or Table **S4** and Figure **2.e** or Table **S5**), which is an absolute improvement in ROC-AUC ranging from 0.7% to 15.3% compared with other methods. This shows that molecular image-based representation has obvious advantages compared with sequence-based molecular representation because these models can only learn 1D sequence information but lacks 2D structural information.

Graph-based models. Considering that molecules can be naturally represented as graphic structures, some graph-based methods [15, 16] have recently emerged to learn the 2D topological structure information of molecules. We compared our ImageMol with several graph-based pre-training model (Jure's GNN) [15]. The performance of ImageMol comprehensively

exceeds the state-of-the-art graph-based pre-training models on all datasets (Figure **2.e** and Table **S5** ranging from 3.4% to 12.6%, which shows the advantage of using molecular images as a representation. Although both molecular graph and image are based on 2D representation, they are significantly different in representation type. The molecular graph focuses on topological information at the atomic level, while the molecular image focuses on the spatial structure information at the pixel level. In spatial structure, more rich information is included, such as the shape of molecules, the angle of chemical bonds, and the relative distance between atoms, etc.

Image-based models. We selected several latest molecular image-based models as comparison methods, which are Chemception, ADMET-CNN and QSAR-CNN respectively. We find that our ImageMol has high performance and outperforms the state-of-the-art methods with a huge performance gap ranging from 6.8% to 37.1% (Figure **2.b,** Figure **2.c** and Figure **S7**). Specifically, we observed a similar performance between ImageMol_NonPretrain and Chemception, which is 73.2% vs 72.2% and 73.4% vs 75.2% on the HIV and Tox21 datasets respectively (Figure **S6**). However, after pre-training on 8M molecular images, our ImageMol showed a significant improvement on the HIV (an increase of 9.9%) and Tox21 (an increase of 7.2%) with an average increase from -0.4% to 8.6%, which proves the effectiveness and superiority of our pre-training strategies for molecular images.

C.3 Results on anti-viral activities across SARS-CoV-2 targets

In Table **S7** and Table **S8**, the experimental results of Jure's GNN, ImageMol and REDIAL-2020 on anti-SARS-CoV-2 activities estimation task are described. In Table **S7**, we obtain the results of Jure's GNN by running its public source code (https://github.com/snap-stanford/pretrain-gnns) on our SARS-CoV-2 dataset. In Table **S8**, REDIAL-2020 announced the dataset they used (https://doi.org/10.5281/zenodo.4606720), including training set, validation set and test set. Therefore, we run ImageMol under the exact same experimental settings as theirs.

C.4 Results on the virtual screening anti-SARS-CoV-2 drugs

Table **S9** shows virtual screening results of approved drugs in DrugBank for 3CL inhibitors. In particular, we performed virtual screening using the 3CL model with 82.4% ROC-AUC in Table **S7**. The predicted label and probability for each drug is in the columns pred_labels, non-inhibitor_probs, inhibitor_probs of Table **S9**. The distribution histogram of predicted 3CL inhibitors is depicted in Figure **S8**. Additionally, to further validate the effectiveness of our approach, we also screened drugs for SARS-CoV-2 from approved drugs. We use HEK293's model for virtual screening because it models larger data volumes and has good performance. The screening results are shown in Table **S11**. The 13 of the top 20 drugs were verified by different literatures, demonstrating the great potential of ImageMol. We also tested the

accuracy of the model on the external validation set (Table **S12**), which is provided by [17] and has 122 inhibitors of the SARS-CoV-2 (shown in https://static-content.springer.com/esm/art%3A10.1038%2Fs41586-022-04482-x/MediaObjects/41586_2022_4482_MOESM1_ESM.pdf). Since we focus on virtual screening of small molecule drugs, we took the intersection of these 122 inhibitors and drugs in DrugBank and finally got 70 small molecules for testing. Of these 70 drugs, we successfully predicted 47 potential drugs, demonstrating the potential of ImageMol as a novels drug discovery tool.

Supplementary Figures

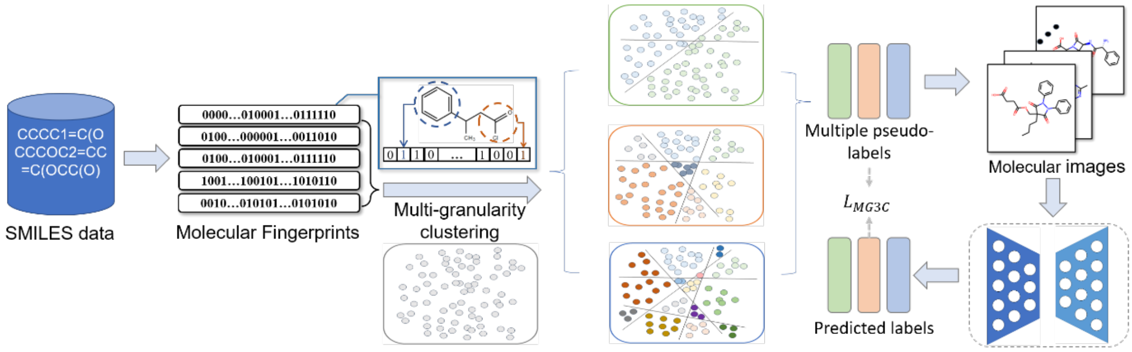

Figure S1: The architectural details of the Multi-Granularity Chemical Clusters Classification (MG3C) task. Firstly, the molecular fingerprints are extracted from SMILES and input into unsupervised multi-granularity clustering to produce clusters with different granularity. Then, these clusters are uniquely numbered as pseudo-labels of molecular images. Finally, the molecular encoder and structural classifier are jointly used to predict the labels of molecular images and optimizing the loss between pseudo-labels and predicted labels in pre-training.

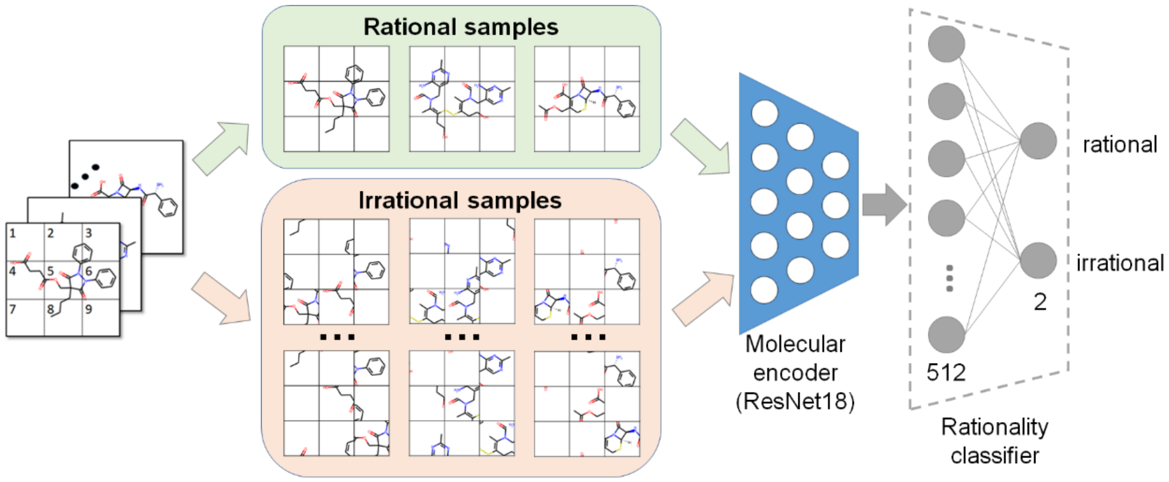

Figure S2: The architectural details of the Molecular Rationality Discrimination (MRD) task. In order to construct an irrational molecular image, we first disrupt the molecular structure, which uses a 3x3 grid to decompose the molecular image into 9 patches and randomly shuffle them to form an irrational image. Then, these rational and irrational molecules will be input to the molecular encoder to extract visual features. Finally, these features are forward propagated to rationality classifier for rationality judgment.

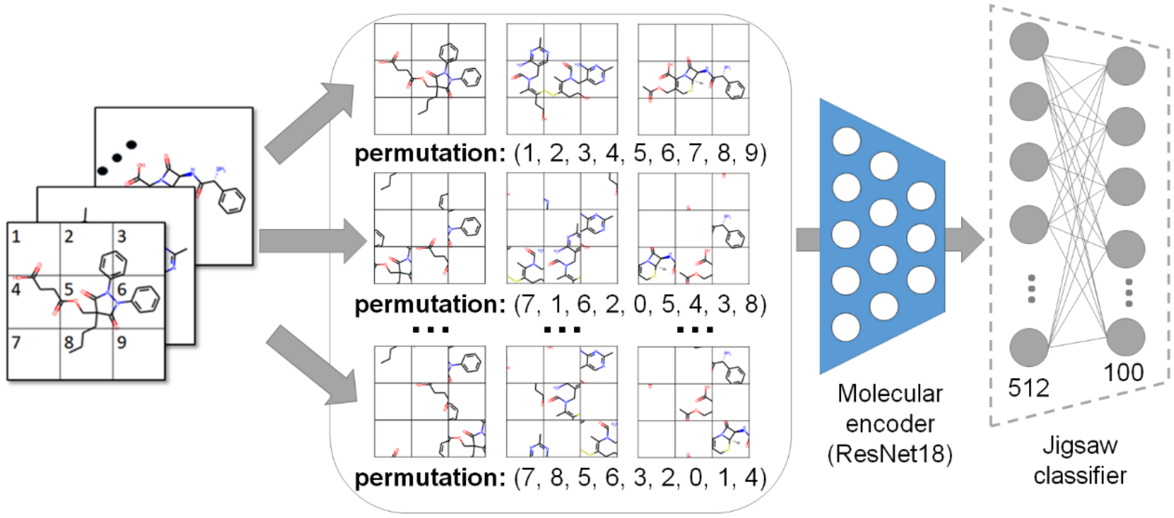

Figure S3: The architectural details of the Jigsaw Puzzle Prediction (JPP) task. We first use a 3x3 grid to decompose the molecular image into 9 patches and assign numbers from 1 to 9. Then, we use different permutations to reorganize the image. Finally, the reorganized images are fed into the molecular encoder and jigsaw classifier to predict the corresponding permutations.

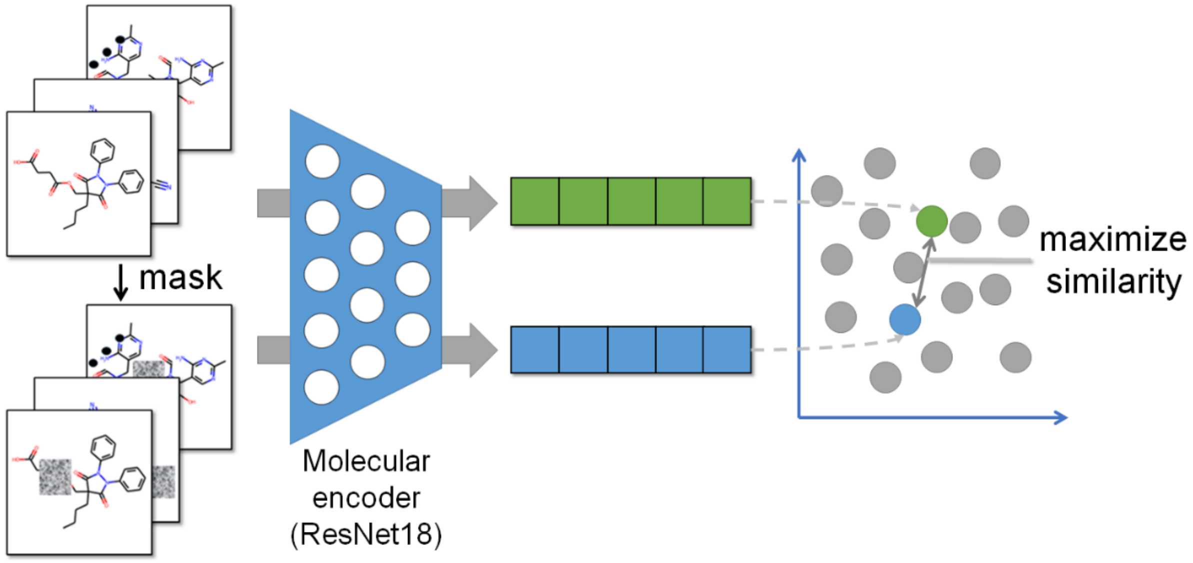

Figure S4: The architectural details of the MASK-based Contrastive Learning (MCL) task. We first randomly mask a 16×16 area to obtain a masked image. Then a pair of images (original image and the masked image) are simultaneously fed into the molecular encoder to extract latent features. Finally, we optimize the molecular encoder by maximizing the similarity among the latent feature pairs.

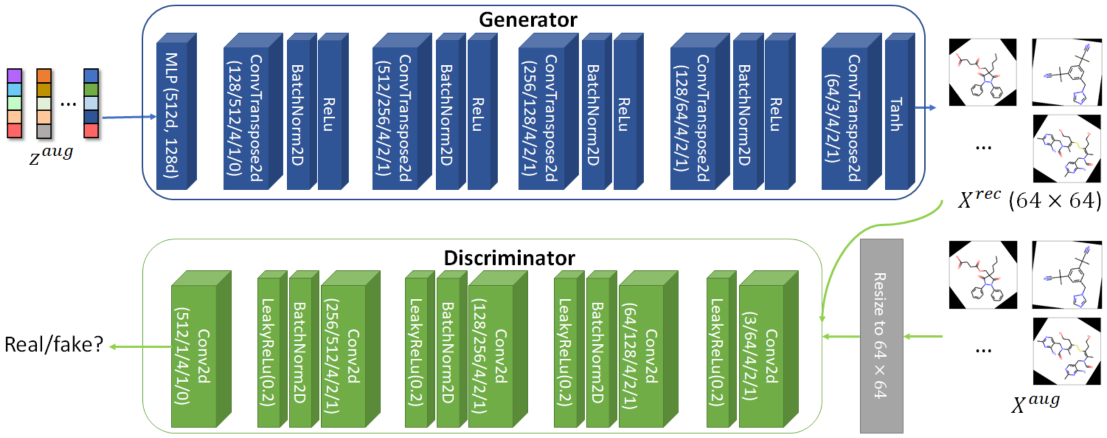

Figure S5: The architectural details of the Molecular Image Reconstruction (MIR) task. The generator is used to reconstruct latent features z^{aug} back into 64×64 molecular images X^{rec}. The discriminator accepts the generated image X^{rec} and the real image X^{aug} and discriminates their real and fake.

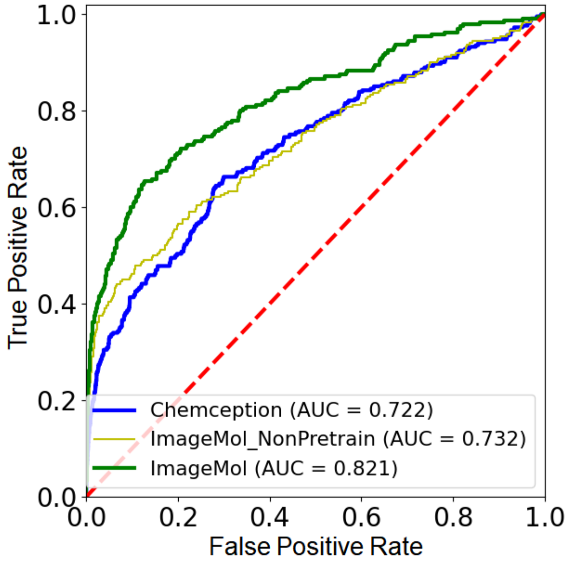

(a) The ROC-AUC curve on HIV dataset

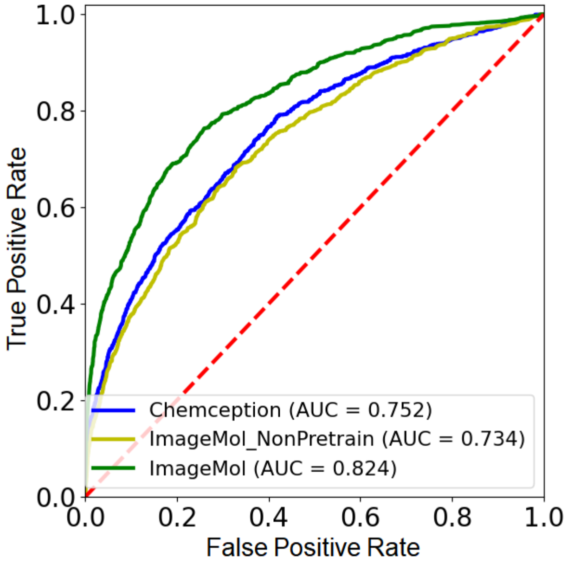

(b) The ROC-AUC curve on Tox21 dataset

Figure S6: Receiver operating characteristic (ROC) curves of Chemception, ImageMol_NonPretrained and ImageMol on Tox21 and HIV datasets. Chemception is the method based on molecular image to predict the molecular property. ImageMol_NonPretrained is the ResNet18 trained from scratch without any pre-training. ImageMol is our pre-trained model based on 8 million molecular images.

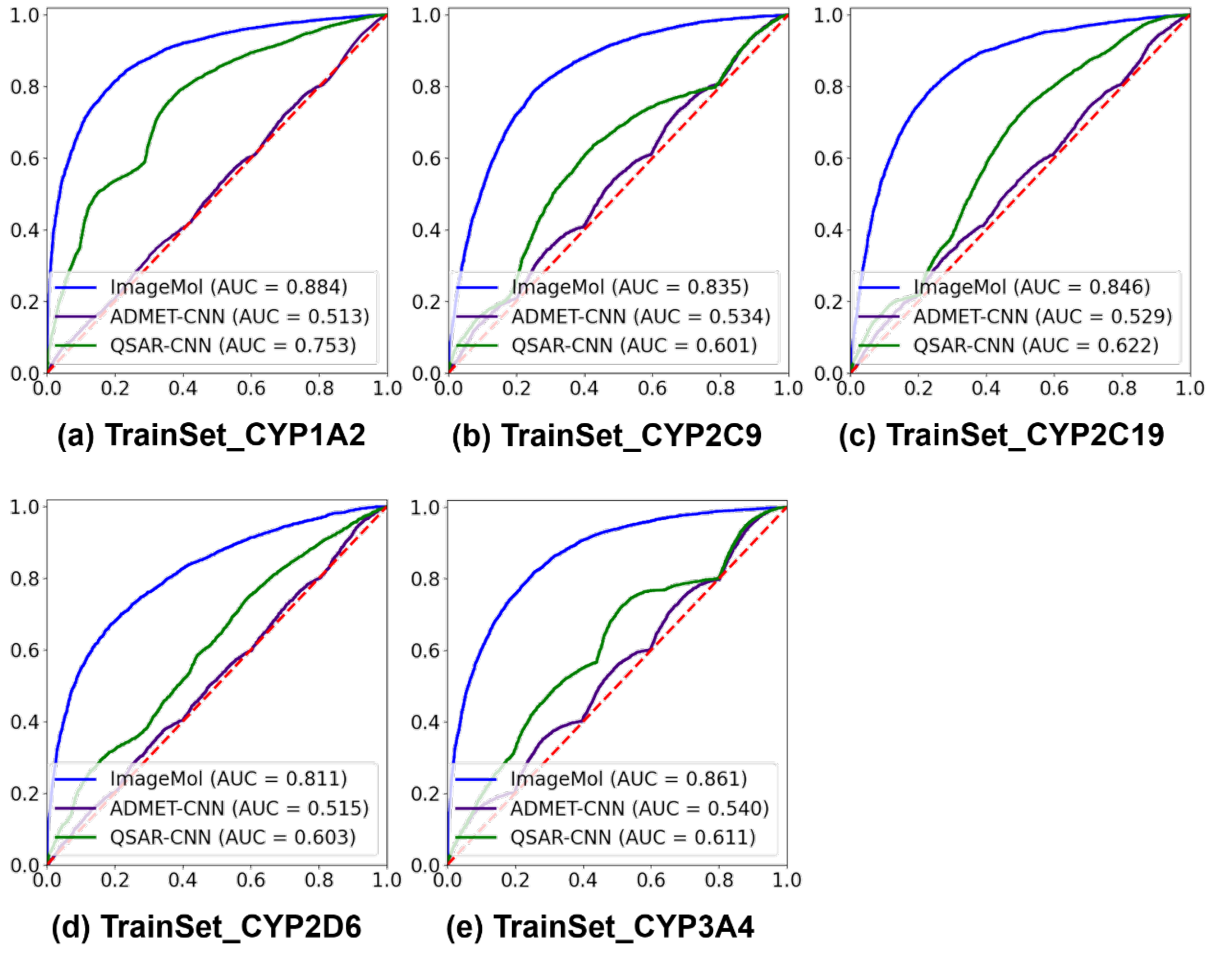

Figure S7: Receiver operating characteristic (ROC) curves of ADMET-CNN [18], QSAR-CNN [19] and ImageMol on five CYP isoforms training sets (PubChem Data Set I) with 5-fold cross-validation.

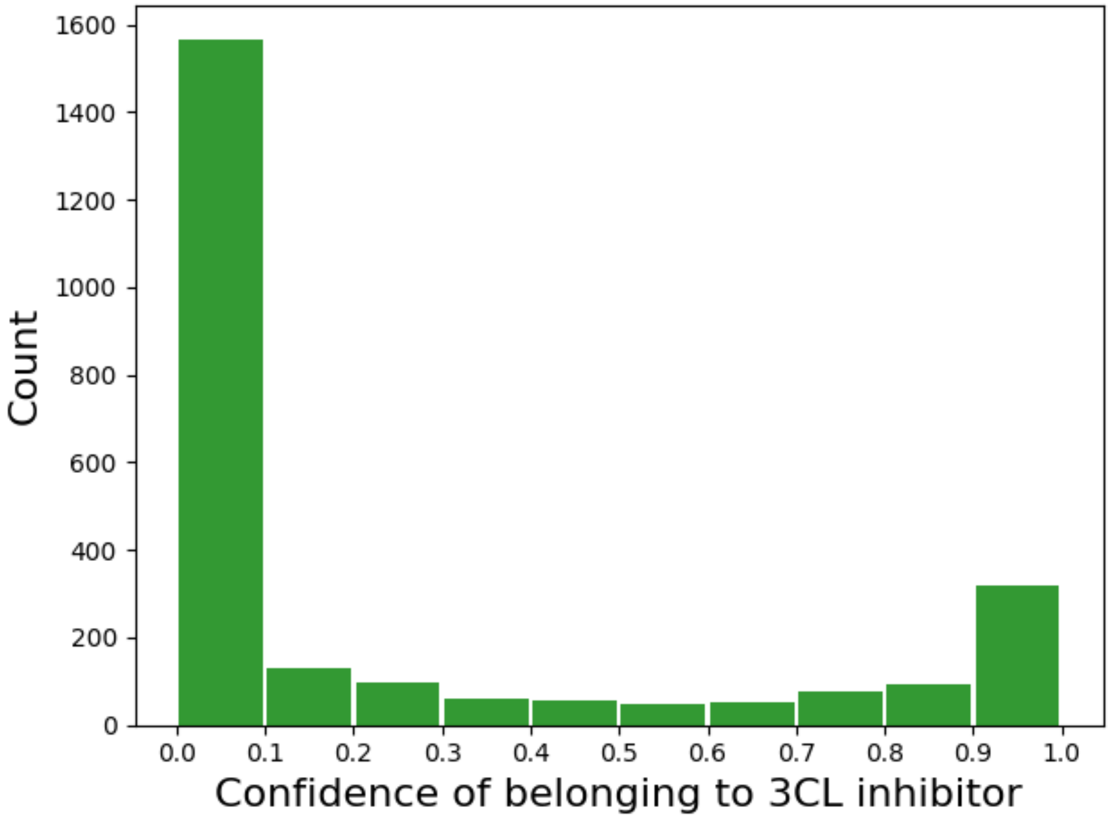

Figure S8: The distribution histogram of drugs that can be used as 3CL inhibitors. The x-axis represents the confidence that the drug is a 3CL inhibitor, and the y-axis represents the number of drugs within a certain confidence interval. The range of confidence is 0 to 1. The higher the confidence, the more likely the corresponding drug is to be a 3CL inhibitor.

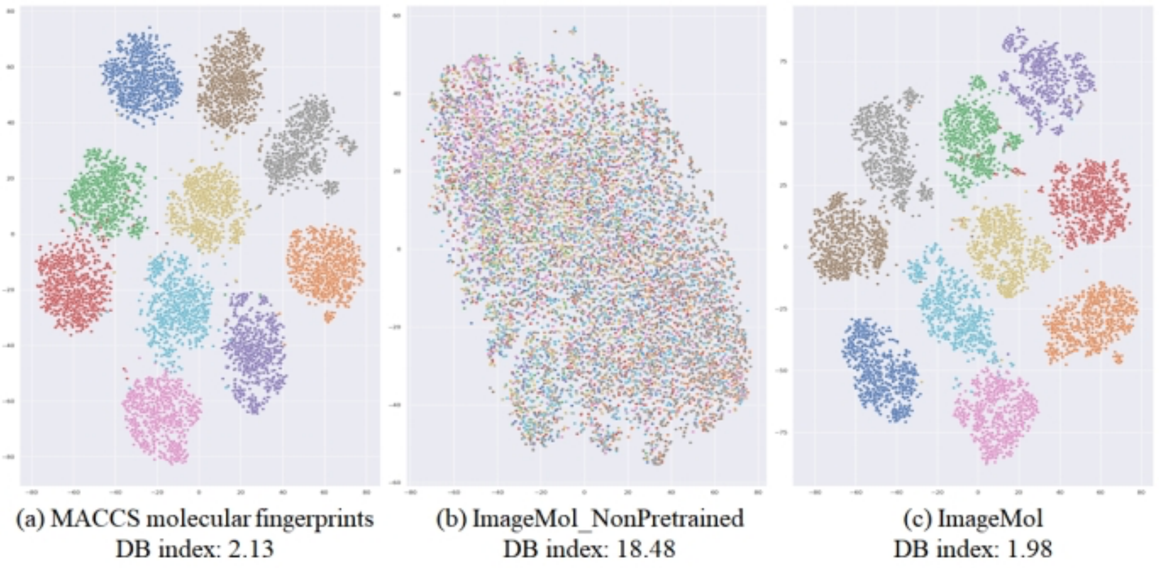

Figure S9: t-SNE visualization of the representations learned by different models. Different colors indicate different clusters. Davies Bouldin (DB) index [20] is defined as the average similarity measure of each cluster with its most similar cluster, where similarity is the ratio of within-cluster distances to between-cluster distances. The lower the DB index value, the better the clustering result.

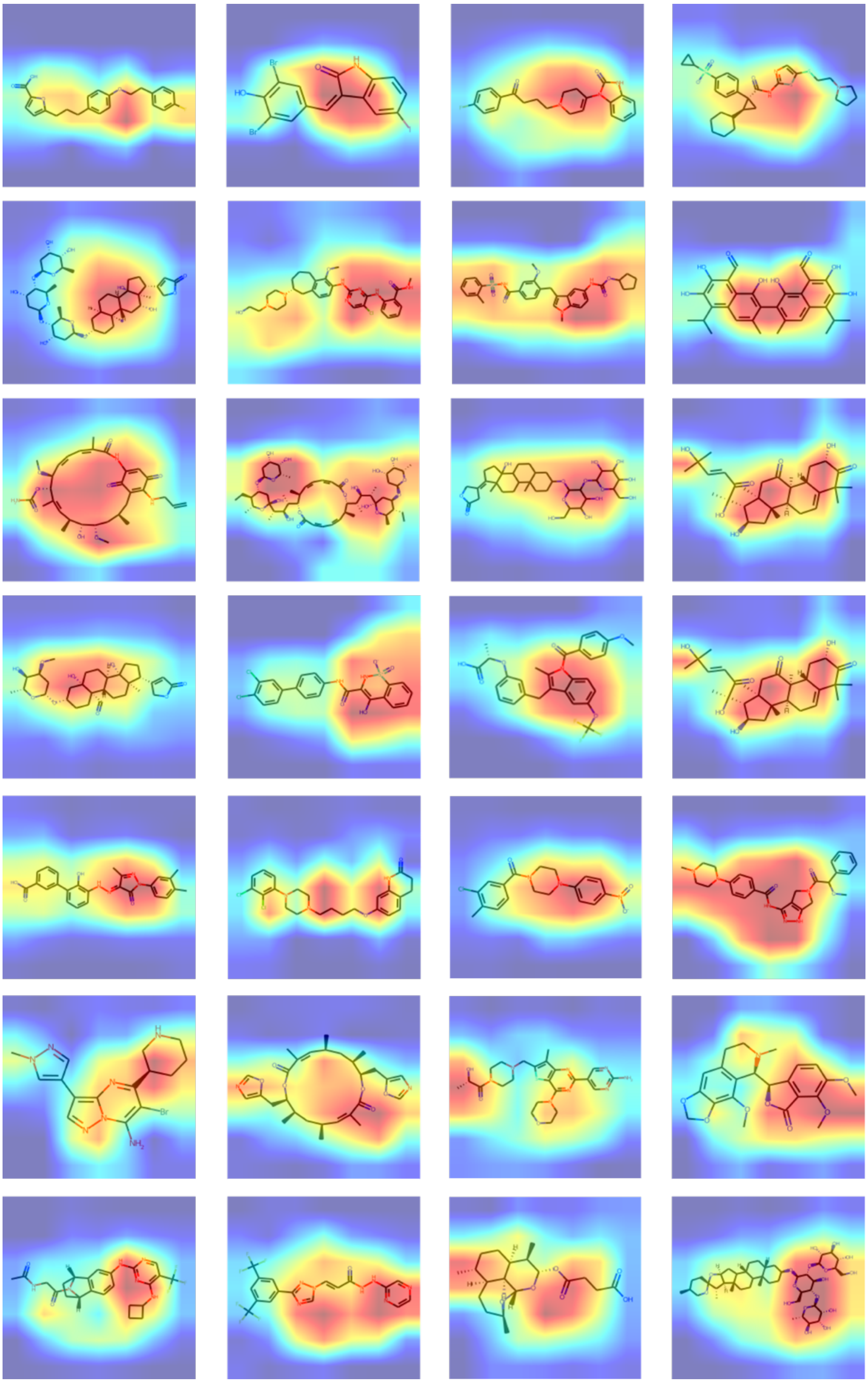

Figure S10: The heat maps of attention to global structural information, which are highlighted by Gradient-weighted Class Activation Mapping (Grad-CAM) [21]. The warmer color, the higher attention; the cooler color, the lower attention. Obviously, all meaningful structural regions are highlighted by ImageMol.

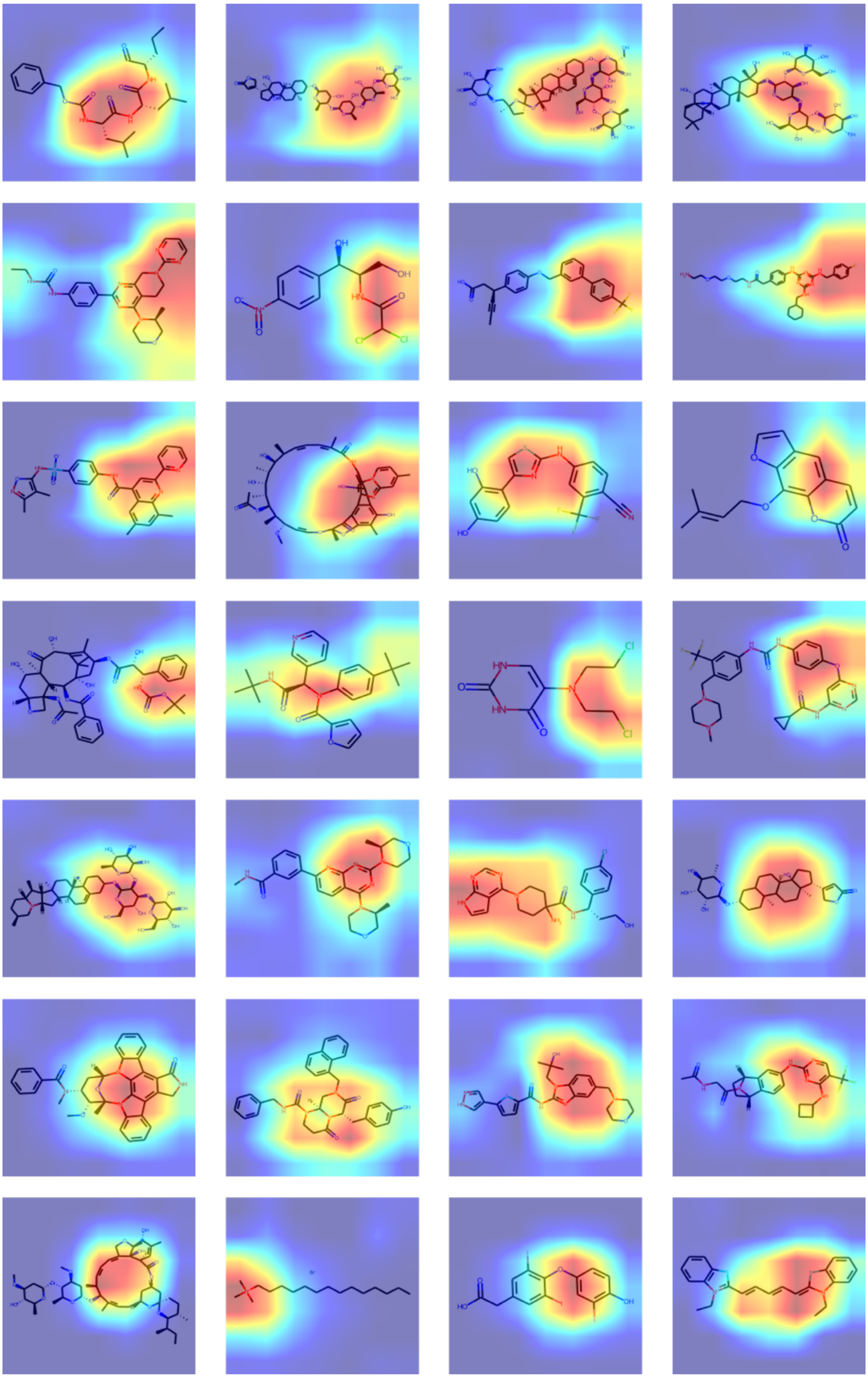

Figure S11: The heat maps of attention to local structural information, which are highlighted by Gradient-weighted Class Activation Mapping (Grad-CAM) [21]. The warmer color, the higher attention, and the cooler color, the lower attention. Obviously, ImageMol captures more local regions for inference.

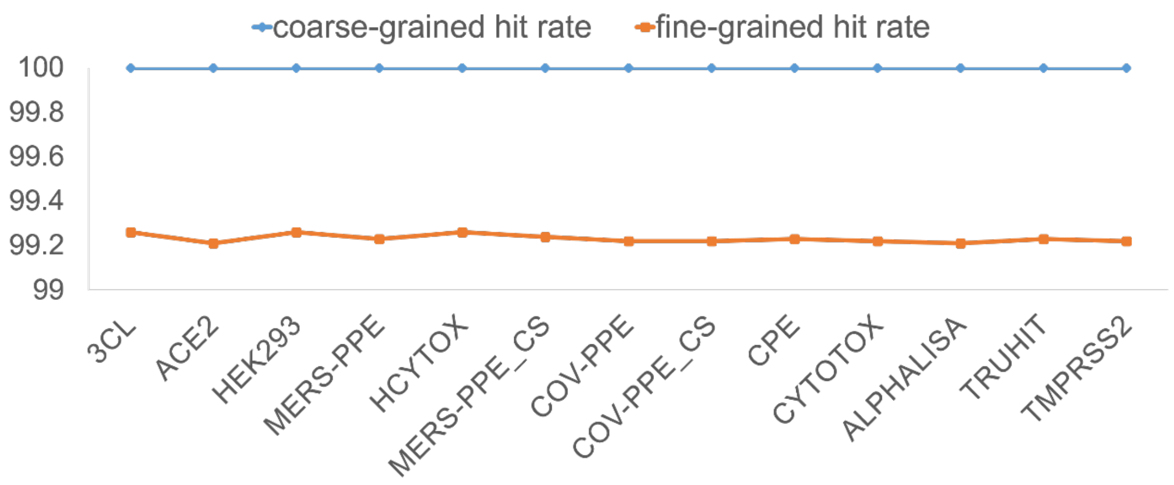

Figure S12: Quantitative information about the molecular structure area that is focused on by ImageMol. The coarse-grained hit rate represents the proportion of the molecular structure in each molecular image that was noticed by ImageMol, and fine-grained hit rate represents the proportion of the molecular structure area that was noticed by ImageMol to the total molecular structure area.

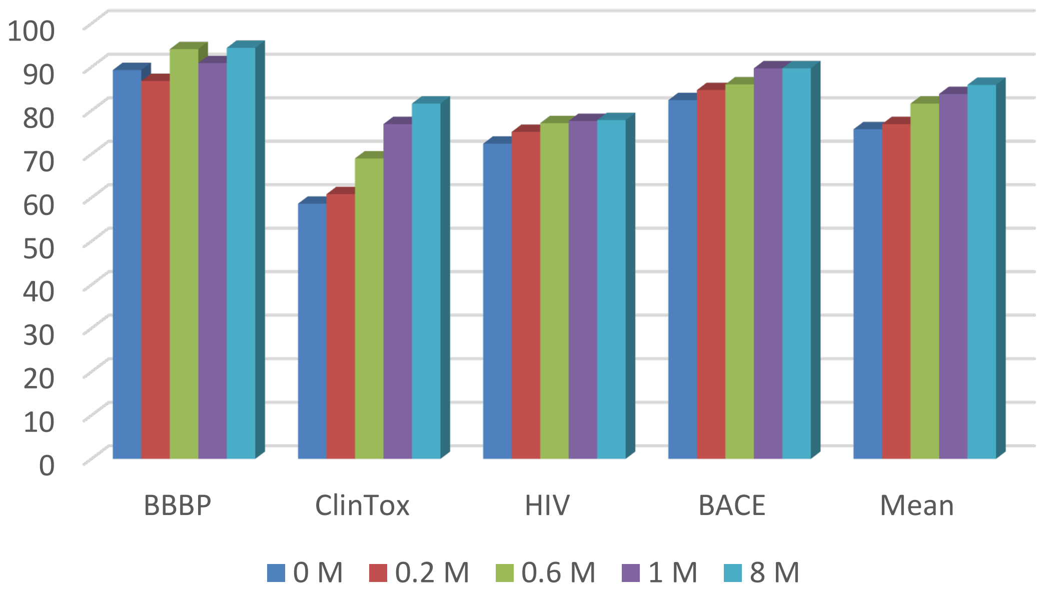

Figure S13: The performance of ImageMol pre-trained under the data scale of 0M (no pre-training), 0.2M, 0.6M, 1M and 8M (our final ImageMol) with scaffold split on four property prediction datasets. “Mean” represents the average performance of ImageMol pre-trained with different data scales on four datasets.

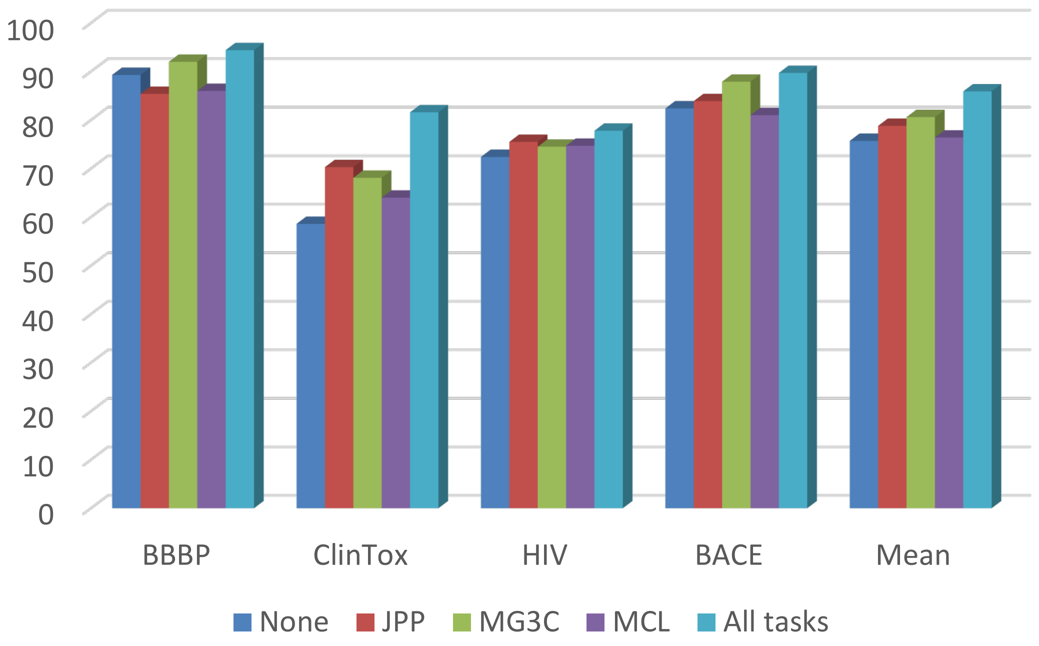

Fig. S14: The ablation study of the pretext task in ImageMol, which uses ROC-AUC evaluation with scaffold split on four property prediction datasets. “Mean” represents the average performance of ImageMol pre-trained with different pre-task on four datasets.

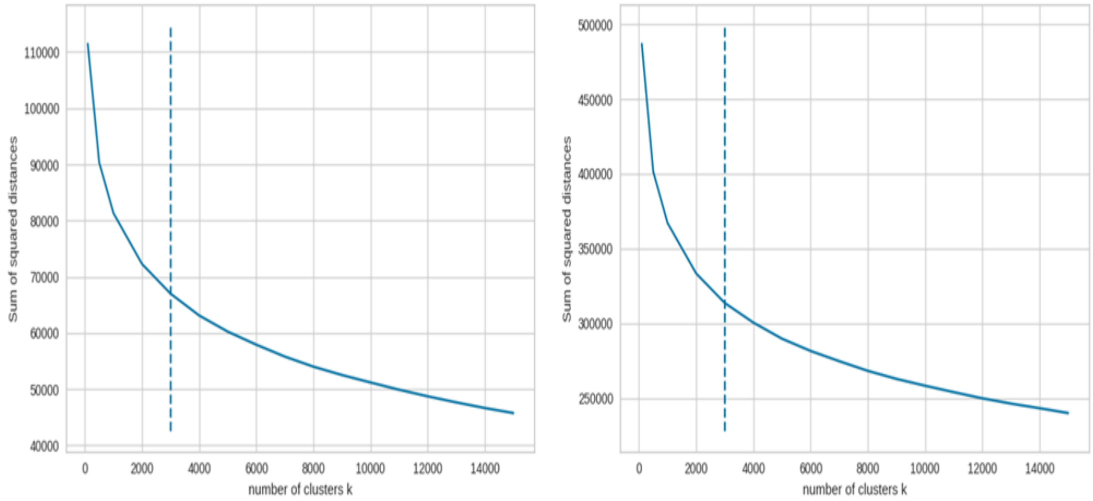

(a) The “elbow” point of ChEMBL dataset

(b) The “elbow” point of ZINC dataset

Figure S15: The “elbow” point of two datasets with respect to the number of clusters. The x-axis represents the number of clusters, and the y-axis represents the sum of Euclidean distances between samples in the clusters. We find the "elbow", which is a value corresponding to the point of maximum curvature in an elbow curve, by using knee point detection algorithm [2].

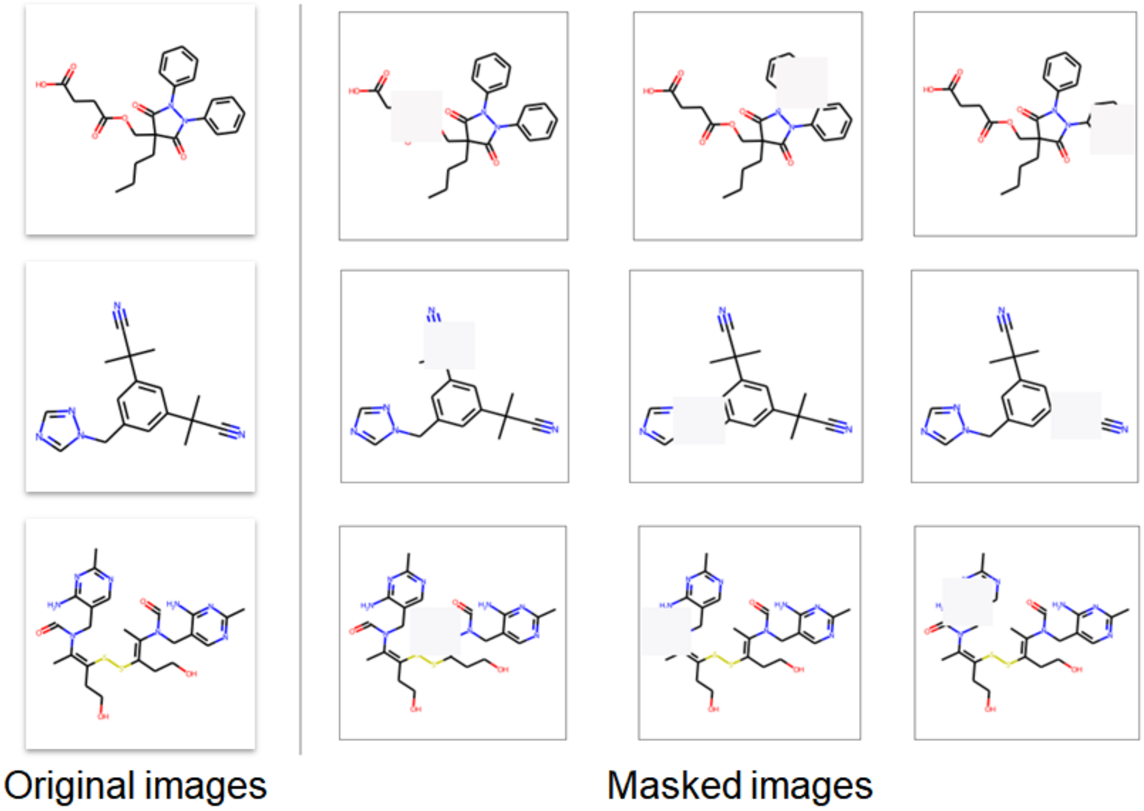

Figure S16: Examples of masked images. The first column represents the original images, and the last three columns represent the masked images.

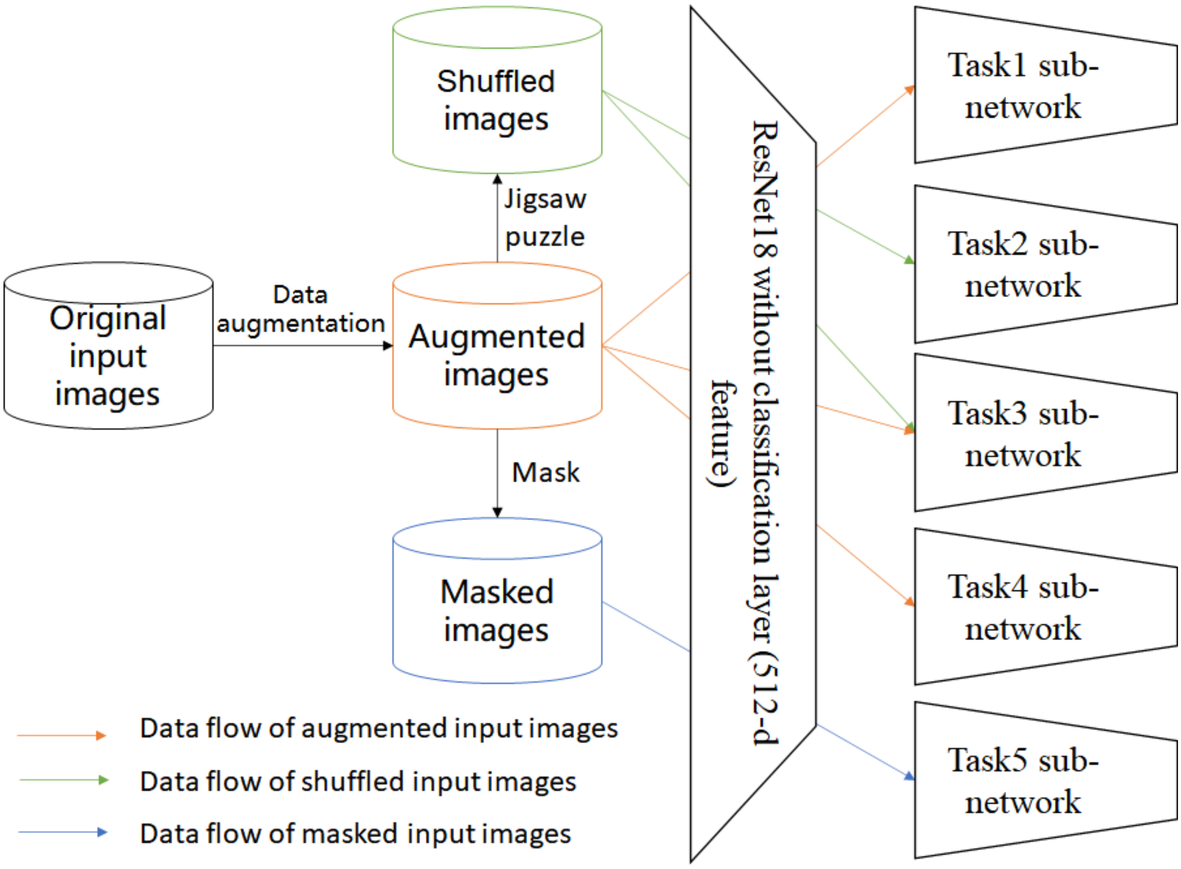

Figure S17: The data flow of the forward propagation of ImageMol framework in pre-training. Data augmentation techniques are first used to extract different augmentations of the original input images and further permutation and masking to obtain shuffled images and masked images, respectively. These images are then fed into ResNet18 to extract latent features. Finally, augmented images are used for task 1, task 3 and task 4. Shuffled images are used in task 2 and task 3. Masked images are used in task 5.

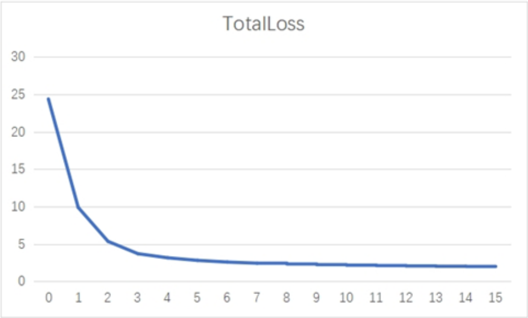

(a) The total loss of ImageMol, which is the sum of the losses of pretext task 1, 2, 3, 4.

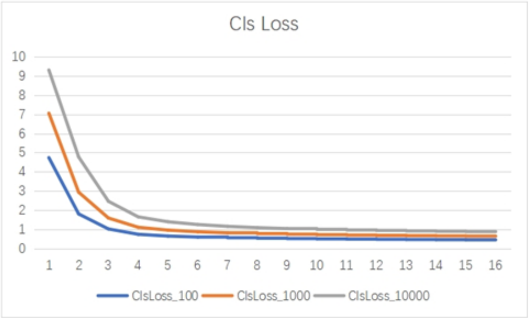

(b) The loss of the pretext task1 during pretraining. ClsLoss_100, ClsLoss_1000 and ClsLoss_10000 represent 100 categories, 1000 categories and 10000 categories.

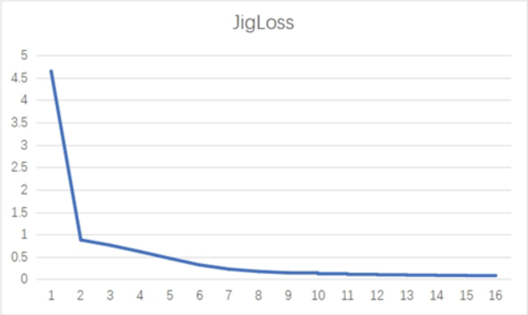

(c) The loss of the pretext task2 during pretraining.

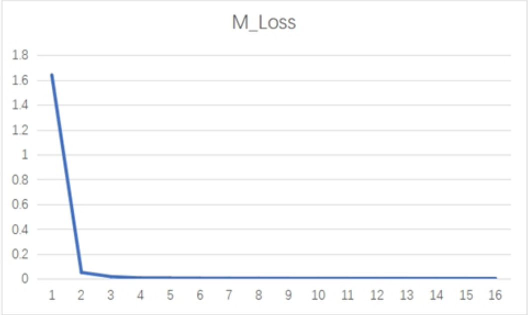

(d) The loss of the pretext task3 during pretraining.

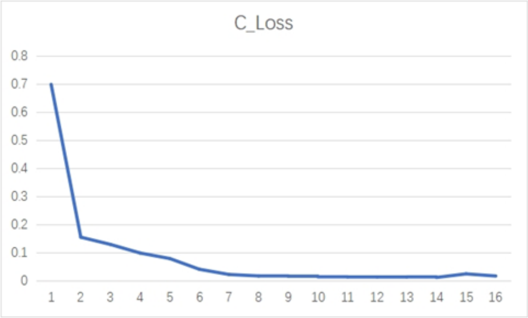

(e) The loss of the pretext task4 during pretraining.

Figure S18: Pre-training details for ImageMol. The x-axis and y-axis represent epoch number and loss value respectively. For simplicity, clustering pseudo-label classification task, jigsaw puzzle prediction task, molecular rationality discrimination task and MASK-based contrastive learning are simplified to pretext task1, pretext task2, pretext task3 and pretext task4 in this group of figures.

Supplementary Tables

Table S1: Basic statistical information of five benchmark datasets about molecular targets (HIV and BACE), blood-brain barrier penetration (BBBP), and drug toxicity (Tox21, ClinTox).

**Dataset**	BBBP	Tox21	ClinTox	HIV	BACE
Molecules	2039	7831	1478	41127	1513
Binary prediction tasks	1	12	2	1	1

Table S2: The statistical information of drug metabolism datasets across 5 main types of CYP450 metabolism enzymes.

<table>
<tr><th>Datasets</th><th>CYP isoforms</th><th>Number of inhibitors</th><th>Number of noninhibitors</th><th>Total</th></tr>
<tr><td rowspan="5">PubChem Data Set I</td><td>1A2</td><td>5663</td><td>6436</td><td>12,099</td></tr>
<tr><td>2C9</td><td>4369</td><td>7761</td><td>12,130</td></tr>
<tr><td>2C19</td><td>5322</td><td>6563</td><td>11,885</td></tr>
<tr><td>2D6</td><td>2516</td><td>9365</td><td>11,881</td></tr>
<tr><td>3A4</td><td>4637</td><td>6899</td><td>11,536</td></tr>
<tr><td colspan="5"></td></tr>
<tr><td rowspan="5">PubChem Data Set II</td><td>1A2</td><td>1752</td><td>1052</td><td>2804</td></tr>
<tr><td>2C9</td><td>609</td><td>1970</td><td>2579</td></tr>
<tr><td>2C19</td><td>719</td><td>1972</td><td>2691</td></tr>
<tr><td>2D6</td><td>544</td><td>2316</td><td>2860</td></tr>
<tr><td>3A4</td><td>2070</td><td>4955</td><td>7025</td></tr>
</table>

Table S3: The accuracy and ROC-AUC value of five major CYP Isoforms from PubChem Data Set II. ImageMol_NonPretrained and ImageMol indicate that ImageMol is not pre-trained and ImageMol is pre-trained on 8M molecular images. "MACCS-" represents the method based on MACCS molecular fingerprint, "FP4-" represents the method based on FP4 molecular fingerprint. "CC-" represents combined algorithm (ensemble learning). For details on other methods, see [7]. ImageMol_NonPretrained and ImageMol are fine-tuned on PubChem Data Set I and evaluated on PubChem Data Set II. The best and second best result for each dataset is bolded and underlined, respectively. (.xlsx)

Table S4: The ROC-AUC (%) performance of different methods on four MPP datasets (BBBP, Tox21, HIV and BACE) with stratified split. ImageMol_NonPretrained and ImageMol respectively represent no pre-training and pre-training on 8M molecular images. The results of ImageMol_NonPretrained and ImageMol are obtaned by using different random seeds to perform 20 times and are reported in the form of mean ± variance. Gain represents the degree of gain of ImageMol compared to methods from other papers. The best result for each dataset is bolded.

**Dataset**	BBBP	Tox21	HIV	BACE
**Sequence-based pre-training methods**				
RNNS2S	88.4	70.9	68.8	72.7
SMILES Transformer	<u>89.5</u>	71.1	69.6	72.0
**Image-based pre-training method**				
ImageMol_NonPretrained	81.9±3.7	<u>73.1</u>±1.4	<u>71.0</u>±2.5	<u>79.1</u>±3.6
ImageMol	**90.2**±1.9	**78.7**±1.2	**79.1**±1.3	**88.0**±1.0
**Gain**	+0.7	+7.6	+9.5	+15.3

Table S5: The ROC-AUC (%) performance of different methods on four MPP datasets (BBBP, ClinTox, HIV and BACE) with scaffold split. ImageMol_NonPretrained and ImageMol respectively represent no pre-training and pre-training on 8M molecular images and their average performance is calculated by using three different random seeds. The results are reported in the form of mean ± variance. Gain represents the degree of gain of ImageMol compared to methods from other papers. The best and the second best result for each dataset is bolded and underlined, respectively.

**Dataset**	BBBP	ClinTox	HIV	BACE
**Graph-based pre-training methods**				
Jure's GNN	91.5±4.0	76.2±5.8	-	85.1±2.7
N-GRAM	91.2±1.3	85.5±3.7	-	87.7±3.5
**Sequence-based pre-training methods**				
ChemBERTa	64.3	73.3	62.2	-
SMILES Transformer	70.4	-	72.9	70.1
Mol2Vec	87.6±3.0	82.8±2.3	-	84.1±5.2
**Image-based pre-training method**				
ImageMol_NonPretrained	79.1±1.3	71.8±4.9	71.6±1.1	82.5±0.7
ImageMol	**94.9**±1.1	**88.8**±2.0	**78.2**±0.6	**89.3**±0.8
**Gain**	+3.4	+3.3	+5.3	+1.6

Table S6: The overview of the processed datasets, which are all binary classification tasks. These datasets are obtained from SARS-CoV-2 assays in NCATS OpenData. Abbreviation represents shorthand for dataset and target category represents the classification type of assay. The number of samples and the number of positive samples are calculated using only samples with AC_{50}.

Dataset (Assay)	Abbreviation	Target Category	Number of samples	Number of positive samples (percentage)
3CL enzymatic activity	3CL	Viral replication	448	84 (18.8%)
ACE2 enzymatic activity	ACE2	Viral entry	650	122 (18.8%)
HEK293 cell line toxicity	HEK293	Counterscreen	4949	1753 (35.4%)
Human fibroblast toxicity	hCYTOX	Counterscreen	5533	2032 (36.7%)
MERS Pseudotyped particle entry	MERS-PPE	In vitro infectivity	6133	2191 (35.7%)
MERS Pseudotyped particle entry (Huh7 tox counterscreen)	MERS-PPE_cs	Counterscreen	6357	2241 (35.3%)
SARS-CoV Pseudotyped particle entry	CoV-PPE	In vitro infectivity	7394	2550 (34.5%)
SARS-CoV Pseudotyped particle entry (VeroE6 tox counterscreen)	CoV-PPE_cs	Counterscreen	7589	2613 (34.4%)
SARS-CoV-2 cytopathic effect	CPE	Live virus infectivity	8430	2937 (34.8%)

(CPE)				
SARS-CoV-2 cytopathic effect (host tox counterscreen)	Cytotox	Counterscreen	10140	3936 (38.8%)
Spike-ACE2 protein-protein interaction (AlphaLISA)	AlphaLISA	Viral entry	11144	4300 (38.6%)
Spike-ACE2 protein-protein interaction (TruHit Counterscreen)	TruHit	Counterscreen	12159	4919 (40.5%)
TMPRSS2 enzymatic activity	TMPRSS2	Viral entry	12357	4944 (40.0%)

Table S7: The experimental results of Jure’s GNN, ImageMol_NonPretrained and ImageMol_8M for anti-SARS-CoV-2 activities estimation on several *SARS-CoV-2 assay* datasets from the National Center for Advancing Translational Sciences (NCATS) COVID-19 portal. The evaluation metrics include the AUC and AUPR. ImageMol_NonPretrained is the ResNet18 randomly initialized weights. ImageMol_8M is our pre-trained model based on ZINC dataset. The best results are bolded and the second best results are underlined. The red value represents the number of performance improvement compared with ImageMol_NonPretrained.

	**AUC**			**AUPR**		
**Dataset**	**Jure’s GNN**	**ImageMol_NonPretrained**	**ImageMol_8M**	**Jure’s GNN**	**ImageMol_NonPretrained**	**ImageMol_8M**
3CL	70.43	76.07	**82.43**$^{+6.36}$	33.96	49.66	**63.10**$^{+13.44}$
ACE2	73.66	65.45	**82.59**$^{+17.14}$	33.56	30.13	**60.41**$^{+30.28}$
HEK293	73.87	70.26	**77.75**$^{+7.49}$	64.32	60.83	**68.67**$^{+7.84}$
hCYTOX	76.19	72.26	**76.56**$^{+9.02}$	**66.19**	63.52	65.24$^{+1.72}$
MERS-PPE	71.71	70.82	**75.20**$^{+4.30}$	60.73	58.78	**65.34**$^{+6.56}$
MERS-PPE_cs	71.19	72.81	**75.96**$^{+3.15}$	57.39	60.96	**66.46**$^{+5.50}$
CPE	69.19	72.06	**74.77**$^{+2.71}$	55.96	58.13	**61.86**$^{+3.73}$
cytotox	72.38	73.88	**75.71**$^{+1.83}$	63.45	65.70	**68.68**$^{+1.97}$
CoV-PPE	70.78	69.95	**74.19**$^{+4.24}$	57.58	57.66	**60.54**$^{+2.88}$
CoV-PPE_cs	70.45	71.60	**74.22**$^{+2.62}$	55.62	58.31	**61.81**$^{+3.50}$
AlphaLISA	69.25	71.28	**73.88**$^{+2.60}$	59.30	62.88	**65.95**$^{+3.07}$
TruHit	67.33	70.19	**72.84**$^{+2.65}$	58.73	61.84	**65.28**$^{+3.44}$
TMPRSS2	67.79	70.09	**71.95**$^{+1.86}$	58.69	61.52	**62.80**$^{+1.28}$

Tab. S8: The experimental results of REDIAL-2020, ImageMol_NonPretrained and ImageMol_8M for anti-SARS-CoV-2 activities estimation. ACC, accuracy; F1, F1 score; SEN, sensitivity; PREC, precision; AUC, area under the receiver operating characteristic curve. (.xlsx)

Table S9: Screening results of approved drugs in DrugBank for 3CL inhibitors via ImageMol. (.xlsx)

Table S10: Screening results from 10 known 3CL inhibitors.

Index	Drug name	Structure	Probability	Evidence
1	Punicalagin	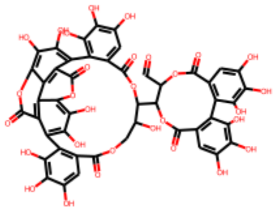	0.9958221	[22]
2	PF-07321332	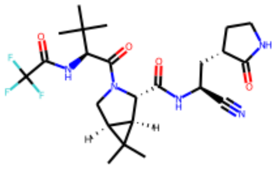	0.97956204	[23]
3	Saquinavir	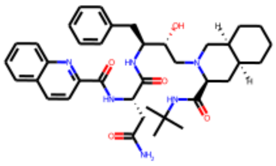	0.73592025	[24]
4	Bafetinib	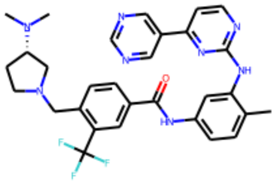	0.6552757	[25]

5	Atazanavir sulfate	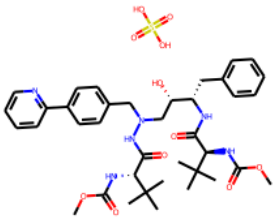	0.616774	[26]
6	Masitinib	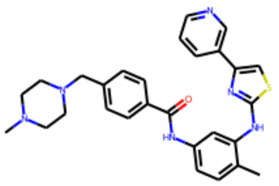	0.6143476	[25]
7	Atazanavir	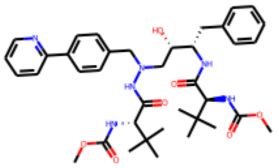	0.2217529	[27]
8	HY-15602	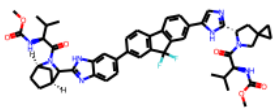	0.1734867	[28]
9	HY-17634	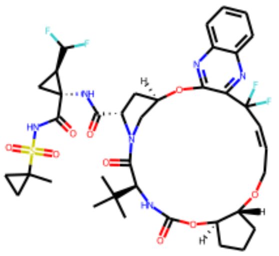	0.0576215	[29]

10	Telaprevir	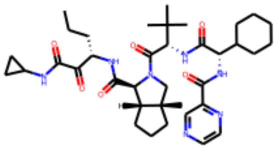	0.05525869	[26]

Table S11: Screening results of approved drugs in DrugBank for SARS-CoV-2. (.xlsx)

Table S12: Virtual screening of 70 validated anti-SARS-CoV-2 small molecule drugs. These drugs were validated in Calu-3 cells [17]. (.xlsx)

Table S13: The statistical information of anti-SARS-CoV-2 activity datasets in [1]. Sparsity refers to the proportion of blank areas in an image to the entire image.

Datasets	Number of actives	Number of inactives	Sparsity (%)
3CL	81	3,330	94.93
CPE	44	2,913	94.89
ACE2	70	1,192	94.88
Cytotox	193	2,764	94.86
AlphaLISA	143	1,119	94.86
TruHit	134	1,128	94.86
CoV-PPE	43	881	94.91
CoV-PPE_cs	247	1,085	94.91

hCYTOX	81	1,306	94.87
MERS-PPE	104	1,024	94.88
MERS-PPE_cs	46	1,082	94.88

Table S14: Hyperparameters for pre-training and finetuning ImageMol.

Hyperparameters	Pre-training	Fine-tuning
Learning rate	0.01	5e-5, 0.005, 0.01
Batch Size	256	8,16,32,64,128
Weight Decay	1e-5	1e-5
Max Epochs	15	60, 120
Learning Rate Decay	Linear	Linear
Image Size	224×224×3	224×224×3
Classification Layer Number	0	1

Supplementary References

1. Bocci, G., et al., *A machine learning platform to estimate anti-SARS-CoV-2 activities.* Nature Machine Intelligence, 2021: p. 1-9.
2. Satopaa, V., et al. *Finding a" kneedle" in a haystack: Detecting knee points in system behavior*. in *2011 31st international conference on distributed computing systems workshops*. 2011. IEEE.
3. Rifaioglu, A.S., et al., *DEEPScreen: high performance drug–target interaction prediction with convolutional neural networks using 2-D structural compound representations.* 2020. **11**(9): p. 2531-2557.
4. Durant, J.L., et al., *Reoptimization of MDL keys for use in drug discovery.* 2002. **42**(6): p. 1273-1280.
5. Carlucci, F.M., et al. *Domain generalization by solving jigsaw puzzles*. in *Proceedings of the IEEE/CVF Conference on Computer Vision and Pattern Recognition*. 2019.
6. Pathak, D., et al. *Context encoders: Feature learning by inpainting*. in *Proceedings of the IEEE conference on computer vision and pattern recognition*. 2016.
7. Cheng, F., et al., *Classification of Cytochrome P450 Inhibitors and Noninhibitors Using Combined Classifiers.* Journal of Chemical Information and Modeling, 2011. **51**(5): p. 996-1011.
8. Arús-Pous, J., et al., *SMILES-based deep generative scaffold decorator for de-novo drug design.* 2020. **12**: p. 1-18.
9. Xue, D., et al., *X-MOL: large-scale pre-training for molecular understanding and diverse molecular analysis.* 2021: p. 2020.12. 23.424259.
10. Xu, Z., et al. *Seq2seq fingerprint: An unsupervised deep molecular embedding for drug discovery*. in *Proceedings of the 8th ACM international conference on bioinformatics, computational biology, and health informatics*. 2017.
11. Honda, S., S. Shi, and H.R.J.a.p.a. Ueda, *SMILES transformer: pre-trained molecular fingerprint for low data drug discovery.* 2019.
12. Chithrananda, S., G. Grand, and B.J.a.p.a. Ramsundar, *ChemBERTa: Large-Scale Self-Supervised Pretraining for Molecular Property Prediction.* 2020.
13. Jaeger, S., S. Fulle, and S. Turk, *Mol2vec: unsupervised machine learning approach with chemical intuition.* Journal of chemical information and modeling, 2018. **58**(1): p. 27-35.
14. Liu, S., M.F. Demirel, and Y. Liang, *N-gram graph: Simple unsupervised representation for graphs, with applications to molecules.* arXiv preprint arXiv:1806.09206, 2018.
15. Hu, W., et al., *Strategies for pre-training graph neural networks.* 2019.
16. Rong, Y., et al., *Self-Supervised Graph Transformer on Large-Scale Molecular Data.* 2020. **33**.

17. Schultz, D.C., et al., *Pyrimidine inhibitors synergize with nucleoside analogues to block SARS-CoV-2.* Nature, 2022: p. 1-9.
18. Shi, T., et al., *Molecular image-based convolutional neural network for the prediction of ADMET properties.* Chemometrics and Intelligent Laboratory Systems, 2019. **194**: p. 103853.
19. Zhong, S., et al., *Molecular image-convolutional neural network (CNN) assisted QSAR models for predicting contaminant reactivity toward OH radicals: Transfer learning, data augmentation and model interpretation.* Chemical Engineering Journal, 2021. **408**: p. 127998.
20. Davies, D.L. and D.W. Bouldin, *A cluster separation measure.* IEEE transactions on pattern analysis and machine intelligence, 1979(2): p. 224-227.
21. Selvaraju, R.R., et al. *Grad-cam: Visual explanations from deep networks via gradient-based localization*. in *Proceedings of the IEEE international conference on computer vision*. 2017.
22. Huang, M., et al., *Punicalagin Inhibited Inflammation and Migration of Fibroblast-Like Synoviocytes Through NF-κB Pathway in the Experimental Study of Rheumatoid Arthritis.* Journal of Inflammation Research, 2021. **14**: p. 1901.
23. Vandyck, K. and J. Deval, *Considerations for the Discovery and Development of 3-Chymotrypsin-Like Cysteine Protease Inhibitors Targeting SARS-CoV-2 Infection.* Current Opinion in Virology, 2021.
24. Fical, L., et al., *Determination of Antiviral Drugs and Their Metabolites Using Micro-Solid Phase Extraction and UHPLC-MS/MS in Reversed-Phase and Hydrophilic Interaction Chromatography Modes.* Molecules, 2021. **26**(8): p. 2123.
25. Drayman, N., et al., *Masitinib is a broad coronavirus 3CL inhibitor that blocks replication of SARS-CoV-2.* Science, 2021. **373**(6557): p. 931-936.
26. Fu, L., et al., *Both Boceprevir and GC376 efficaciously inhibit SARS-CoV-2 by targeting its main protease.* Nature communications, 2020. **11**(1): p. 1-8.
27. Sun, Q., et al., *Bardoxolone and bardoxolone methyl, two Nrf2 activators in clinical trials, inhibit SARS-CoV-2 replication and its 3C-like protease.* Signal transduction and targeted therapy, 2021. **6**(1): p. 1-3.
28. Bobardt, M., et al., *The combination of the NS5A and cyclophilin inhibitors results in an additive anti-HCV inhibition in humanized mice without development of resistance.* Plos one, 2021. **16**(5): p. e0251934.
29. Vevea, J.D. and E.R. Chapman, *Acute disruption of the synaptic vesicle membrane protein synaptotagmin 1 using knockoff in mouse hippocampal neurons.* Elife, 2020. **9**: p. e56469.
